# Stroma-driven horizontal transfer of TCA-related proteins mediates metabolic plasticity and imatinib resistance in chronic myeloid leukemia

**DOI:** 10.1186/s12964-025-02564-7

**Published:** 2025-12-02

**Authors:** Piotr Chroscicki, Nikodem Kasak, Dorota Dymkowska, Laura Turos-Korgul, Dominik Cysewski, Vira Chumak, Dawid Stepnik, Monika Kusio-Kobialka, Agata Kominek, Magdalena Lebiedzinska-Arciszewska, Alicja Krop, Joanna Szczepanowska, Mariusz Wieckowski, Tomasz Stoklosa, Krzysztof Zablocki, Katarzyna Piwocka

**Affiliations:** 1 Laboratory of Cytometry, Nencki Institute of Experimental Biology, Polish Academy of Sciences, Warsaw, Poland; 2 Laboratory of Cellular Metabolism, Nencki Institute of Experimental Biology, Polish Academy of Sciences, Warsaw, Poland; 3 Clinical Research Centre, Medical University of Bialystok, Bialystok, Poland; 4 Laboratory of Mitochondrial Biology and Metabolism, Nencki Institute of Experimental Biology, Polish Academy of Sciences, Warsaw, Poland; 5 Laboratory of Genetics, University Clinical Center, Medical University of Warsaw, Warsaw, Poland; 6 Department of Tumor Biology and Genetics, Medical University of Warsaw, Warsaw, Poland

**Keywords:** Leukemia microenvironment, Bone marrow stroma, TNTs, TCA, Glutamine, Mitochondria, Metabolic plasticity, Proteome, Resistance, Imatinib

## Abstract

**Background:**

Alterations in cancer cell metabolism have recently gained considerable attention as a possible cause of adaptation and resistance to therapy. However, the underlying molecular mechanisms, particularly in leukemia resistance occurring in the bone marrow microenvironment, remain unclear. Here, we explore the role of direct stroma-leukemia interactions and transfer of membrane vesicles along with proteins as a mechanism of stroma-driven protection.

**Methods:**

K562 CML leukemia cells and primary CD34 + CML blasts were cultured alone or co-cultured with HS-5 stromal cells to mimic the bone marrow microenvironment conditions. Imatinib treatment was used experimentally as it is a standard first-line treatment in CML. Assessment of vesicles transfer, metabolic parameters, mitochondrial function phenotyping, Trans-SILAC proteomics and metabolomics, together with apoptosis assessment, verified the influence of stroma on metabolic plasticity, protein transfer and adaptation to imatinib in leukemic cells. Trans-system evaluated necessity of direct cell-cell contact. Data from single-cell atlas of diagnostic CML bone marrow were used to correlate gene expression profiles with clinical outcome. Telaglenastat was used to validate the clinical potential of our findings.

**Results:**

Stromal cells enhanced metabolic plasticity and oxidative capacity in leukemia, thereby protecting against metabolic decline and oxidative stress caused by imatinib. Direct stroma-leukemia contact was necessary for vesicles transfer, metabolic rearrangement and protection from imatinib-induced apoptosis. This was accompanied with shift towards OXPHOS activity, associated with increased utilization of non-glucose substrates. We found the presence of stromal TCA-related proteins in leukemic cells, associated with higher TCA cycle dynamics and activity, increased glutamine and reduced oxidative stress. The gene expression profiles correlated with clinical resistance to TKIs. Targeting the glutamine-TCA axis by telaglenastat in combination with imatinib reversed the stroma-driven protection, leading to increased apoptosis.

**Conclusion:**

This study describes a novel mechanism of direct bone marrow-mediated protection of leukemic cells from imatinib/TKI, related to transfer of metabolic proteins leading to higher activity of TCA cycle, metabolic plasticity and adaptation. Targeting the stroma-driven TCA cycle-related metabolism combined with imatinib presents a promising strategy to achieve therapeutic efficacy to overcome bone marrow microenvironment-mediated protection in CML.

**Supplementary Information:**

The online version contains supplementary material available at 10.1186/s12964-025-02564-7.

## Introduction

Myeloid leukemias represent a complex disease in which leukemic cells exist in various microenvironmental niches, such as the bone marrow (BM) and peripheral blood. Therefore, therapeutic strategies should effectively eliminate malignant cells from these different microenvironments to achieve a favorable clinical outcome. However, growing evidence suggests that leukemia cells, including leukemia cells residing in the bone marrow are resistant to currently available therapies, leading to relapse [1]. Therefore, even if a significant reduction in the circulating population of chronic myeloid leukemia (CML) cells is observed after treatment with tyrosine kinase inhibitors, minimal effects are usually noticed in the BM-residential blasts [2]. Further studies, including our own, demonstrated that bone marrow stromal cells contribute to resistance to apoptosis in both CML and acute myeloid leukemia (AML) cells [3–6]. Overall, it is clear that the bone marrow microenvironment plays a key role in protecting leukemic cells and limiting their eradication, which influences the efficacy of therapy. In this regard, the bone marrow microenvironment (BMM) has been recognized as a critical factor in preclinical drug screening for effective therapy [7–9]. However, the molecular mechanisms responsible for BMM-induced leukemia resistance, as well as possible novel therapeutic targets and strategies to overcome this protection, are still far from being defined.

Emerging evidence shows that the BM stromal cells interact with leukemia cells, both directly and indirectly. However, the precise mechanisms remain partially unclear. One of the recently investigated mechanisms of the intercellular cross-talk is direct cell-to-cell connection, which facilitates the transfer of membrane vesicles, organelles, and proteins between cells through the tunneling nanotubes (TNTs). The identification of this mechanism was a critical turning point in the understanding of intercellular communication. It indicated formation of the cellular network able to directly transfer various cargos between distant cells [10, 11], and revolutionized also our thinking about the leukemia BMM.

Cancer cells can utilize TNTs to transfer the information within the microenvironment, to immune cells or BM niche [12]. Our study indicated that TNTs are formed between stromal and leukemic cells and mediate the transfer of membrane vesicles along with proteins towards leukemia cells. This led to protection from apoptosis after treatment with tyrosine kinase inhibitor (TKI) imatinib [5]. Other groups have also reported the formation of TNTs between different types of leukemia cells and cells within the microenvironment [12, 13]. This included studies which identified communication mechanism by TNTs between ALL cells and mesenchymal stromal cells, leading to bone marrow-mediated resistance to prednisolone [13]. Collectively, these findings emphasize the role of TNTs as potential mediators of the stroma-mediated protection.

Growing evidence points to reprogramming of metabolic activity, including metabolic shifts such as altered glucose and glutamine/amino acids utilization, the Warburg Effect and oxidative metabolism as mechanisms mediating cancer survival and drug resistance [14, 15]. Given the role of mitochondria as energetic hub of the cell, the capacity for metabolic adaptation which relies on the mitochondrial reserve to meet increasing energy needs is often critical for development of resistance. In this context, the tricarboxylic acid (TCA) cycle and oxidative phosphorylation (OXPHOS) work together to metabolize fatty acids, glucose, and amino acids, which feed the TCA cycle. Among the various mitochondrial respiratory parameters, the spare respiratory capacity (SRC) is a particularly valuable measure that indicates the functional mitochondrial reserve, allowing for energetic adaptation. An adaptive metabolic response is often observed in leukemia and specifically in leukemia stem cells (LSCs) treated with chemotherapeutics [14–16]. Thus, mitochondrial metabolic plasticity seems to be a key factor for cancer survival and the development of resistance. Recent studies have highlighted the transfer of the whole mitochondria between distant cells, to support oxidative phosphorylation, ATP production, and the overall metabolic state [5, 17, 18]. However, the direct transfer of membrane vesicles and functional mitochondrial proteins between distant cells has never been investigated as a possible mechanism mediating the reprogramming of metabolism and metabolic plasticity in cancer cells.

To evaluate the role of BM stroma in metabolic reprogramming and protection of leukemia, a co-culture of CML cell line or primary CD34 + leukemic cells with stromal HS-5 cells, which mimics the BMM, was introduced. Treatment with imatinib (1st generation TKI and 1 st line treatment in CML) allowed to study the mechanism of the stroma-mediated resistance.

Together, we discovered a previously unknown stroma-mediated mechanism that protects leukemic cells from TKIs via direct transfer of membrane vesicles along with TCA cycle-associated proteins from stromal to leukemic cells. In particular, it increases metabolic plasticity in leukemia and prevents the metabolic decline caused by the drug treatment. Combination of telaglenastat with imatinib reversed the BM-mediated protection and increased apoptosis in leukemic cells. These findings position targeting of the TCA cycle-related metabolism as possible therapeutic intervention to reverse the BMM-driven protection and effectively eliminate leukemic cells from the bone marrow in myeloid leukemias.

## Methods

### Cell lines and co-culture system

Human bone marrow mesenchymal stromal (HS-5; ATCC#CRL-11882) and human chronic myeloid leukemia (K562; ATCC#CCL-243) cell lines were used to model the stroma-leukemia interaction. Cells were routinely tested for mycoplasma contamination. The mono- and co-cultures were grown in IMDM culture medium (Biowest, #L0191) supplemented with 10% [v/v] fetal bovine serum (Biowest, FBS, #S1819) and 1% [v/v] penicillin (100 U/mL) and streptomycin (100 µg/mL) (Biowest, #L0022) in advised conditions as described [5]. Briefly, 24 h before co-culture, HS-5 cells (1 × 10^6^) were seeded to 5 ml medium in T25 culture flask. After 24 h HS-5 cells had 60–70% of confluence. If needed flow cytometric separation of both cell types, K562 cells were labeled with 20 µM eBioscience Cell Proliferation Dye eFluor450 or Cell Proliferation Dye eFluor670 (Invitrogen by Thermo Fisher Scientific), in cell culture medium according to the manufacturer’s instructions. For co-culture, medium from HS-5 cells was removed, and K562 cells (1 × 10^6^ in 5 ml of fresh medium) were added to HS-5 culture. All the analyses were conducted on leukemic cells after 24-hour-incubation of the co-culture. In co-culture conditions, if not sorted as indicated, K562 suspension cells were carefully harvested after gentle mixing the co-culture to detach leukemic cells from stroma. The contamination with floating HS-5 cells was verified by flow cytometry in co-cultures with both cell types tracked with fluorescent dyes or GFP and did not exceed 0.5%. To physically separate donor HS-5 and recipient K562 cells, a trans-well system (ThinCert, Greiner Bio-One), 1 μm pores, 2 × 106 pores/cm2 was used. Conditioned medium (CM) was collected after 24 h of HS-5 cell culture, centrifuged to remove cells and cellular debris, and added to acceptor cells in 12-well culture plates. HS-5-DLST-GFP cell line was generated using lentiviral vector encoding the DLST gene (Origene, #RC201220L4V) in the presence of 8 µg/mL polybrene (Santa Cruz Biotechnology, #sc-134220). 72 h post-transduction cells were selected with 0.5 µg/mL puromycin for 8 days. The population highly expressing the DLST protein (GFP-positive) was sorted on Cytek Aurora CS Cell Sorter.

### Treatment

Imatinib (generous gift from Pharmaceutical Institute in Warsaw) was added to a final concentration of 1 µM. Telaglenastat (CB-839) (Selleck Chemicals, Houston, USA) was used in the final concentration of 1 µM or 10 µM.

### Generation and visualization of rho0 cells

K562 rho0 (ρ0) cells were generated by culture in IMDM culture medium (originally supplemented with 110 mg/l Sodium Pyruvate) with 10 µM ddC (zalcitabine, ZellBio GmbH, #T0120) to the IMDM culture medium. After 72 h, culture medium was replaced with a fresh portion of 10 µM ddC and incubated for another 48 h. Such prepared cells deprived of mtDNA were used for further experiments. Imatinib was added to a final concentration of 1 µM during the 24 h of co-culture.

Cells seeded on 12 mm diameter glass coverslips were fixed with 4% paraformaldehyde in PBS for 15 min at room temperature, rinsed with 10% FBS/PBS then, coverslips were mounted on glass slides with anti-fading mounting medium coating (Dako, Carpinteria, CA, USA). Fluorescence of mito-GFP was visualized with a Zeiss LSM 780 (Carl Zeiss, Oberkochen, Germany) confocal microscope equipped with a 63x oil immersion objective. Images were acquired at the maximal resolution (pixel size ~ 0.07 × 0.07 μm) and the optimal Z-stack step of 0.39 μm throughout the Z-plane, from randomly selected fields of non-confluent cells. Imaging conditions (gain levels, confocal aperture size and laser power) were held constant in a series of images and the fast scan option was used to minimize bleaching and phototoxic effects. All images were processed and analyzed using Zeiss LSM780 software.

#### Isolation of CD34 + CML patient cells

Patients’ material was obtained from the Department of Hematology, Transplantation and Internal Medicine, Medical University of Warsaw, following informed written consent, in accordance with the Declaration of Helsinki and the guidelines for good clinical practice. All protocols were approved by the local Ethical Committee (Ethical and Bioethical Committee UKSW, Approval No. KEiB-19/2017, Approval No.WAW2/059/2019 and WAW2/51/2016). Peripheral blood mononuclear cells (PBMC) were isolated from two chronic myeloid leukemia patients diagnosed in chronic phase (before any treatment) by density gradient centrifugation. All experiments were performed on the pure population of CD34 + CML blasts. The CD34 + cells were separated using EasySep human CD34 + selection cocktail (StemCell Technologies, Inc.), and the pure CD34 + population was used for experiments. CD34 + cells were short-term cultured as monoculture or co-culture with HS-5 stromal cells in StemProTM-34 SFM medium with nutrient supplement (40x), 1% penicilin-streptomycin, 1% L-glutamine, 50 ng/ml of stem cell factor (SCF), 50 ng/ml of FLT3 ligand, 50 ng/ml of thrombopoietin (TPO), 10 ng/ml of interleukin 3 (IL-3), 10 ng/ml of granulocyte-macrophage colony-stimulating factor (GM-CSF) and 5 ng/ml of interleukin 6 (IL-6). Patients’ characteristics are presented in Supplementary Table 1.

### Flow cytometry

Apoptosis was assessed in leukemic cells growing in monoculture or co-culture, untreated or treated with imatinib for 24 h and stained with AnnexinV-PE and 7-AAD (BD Pharmingen, # 559763) according to the manufacturer’s instructions.

For flow cytometric separation of both cell types, acceptor K562 cells were additionally labeled before co-culture with 20 µM eBioscience Cell Proliferation Dye eFluor 450 (Invitrogen by Thermo Fisher Scientific), or Dye eFluor670 (Invitrogen by Thermo Fisher Scientific), as indicated, according to the manufacturer’s instructions.

To assess mitochondrial transfer, the flow cytometric analysis of stromal GFP-tracked mitochondria uptake by K562 cells was performed. The percentage of GFP-positive leukemic cells was assessed.

For flow cytometry BD LSRFortessa cytometer was used, followed by data analysis using Diva and FlowJo v10.8 software (BD Life Sciences).

### Exchange of membrane vesicles between cells

Donor cells (HS-5) were labeled with DiD Cell-Labeling Solution (catalog no. V22887, ThermoFisher Scientific) as described [5]. Working solution containing 1.5 µl of DiD dye/1 mL of cell culture medium was added to cells for 15 min at 37 °C. Then, HS-5 and K562 cells were seeded in co-culture as described in detail above.

### Exchange of mitochondria between cells

To analyze mitochondria transfer, HS-5 cells were transduced with rLV.EF1.AcGFP1-mito-9 lentiviral vector (TaKaRa) for stable mitochondria labeling. Afterwards, HS-5-mitoGFP were seeded in co-culture with acceptor K562 cells as described in detail above.

### Oxygen consumption

Cellular respiration was measured in lymphocytes after mono- or co-culture, using Oxygraph-2k (OROBOROS^®^INSTRUMENTS GmbH, Austria) at 37 °C. 1 mln of cells was resuspended in PBS containing respiratory substrates (1 mM pyruvate, 5 mM glutamine, 25 mM glucose). Oligomycin (0.1 µg/ml) and CCCP (1 µM) were added sequentially. The respiration rate was normalized to the amount of protein (number of cells) in the assay.

### Lactate production

Cell extracts were prepared in the same manner as for the ATP content analysis. Lactate content was measured in the neutralized (2 M K2CO3) extracts fluorometrically using the standard enzymatic Lactate Assay Kit with lactate dehydrogenase (#MAK570, Sigma Aldrich) in a buffer containing: 0.4 M hydrazine sulfate, 0.5 M glycine, 5 mM EDTA; pH 9.5 and NAD 25 mg/mL (all from Sigma Aldrich). All data were normalized according to the protein content and presented as lactate concentration in nanomoles per milligram of total protein.

### Seahorse analysis

20 µL/well of 10% poly-L-lysine (PLL; Sigma, cat.P8920-100ML) solution was applied on the Agilent Seahorse culture plate (cat. 103793-100). After 20 min incubation at 37 °C, the wells were washed and left to dry overnight at 37 °C. K562 cells were collected from monoculture or co-culture after gentle removing suspension cells, spun and resuspended in Seahorse XF DMEM bicarbonate-free culture medium (pH 7.4; 103575-100, Agilent Technologies, Inc.). 0.2 mln cells/well were seeded onto a PLL-coated Seahorse culture plate. Next, the plate was centrifuged to attach the cells to the PLL surface and incubated at 37 °C, 0% CO2, atmospheric oxygen for 40 min. Oxygen consumption rate (OCR) was measured with the Agilent Seahorse XF Analyzer. Standard Mitochondrial Stress Test was performed according to the manufacturer’s instructions. The modulators of respiration were added as follows: oligomycin 1.5 µM, FCCP 1 µM, rotenone and antimycin A 0.5 µM (Sigma Aldrich). Simultaneously, extracellular acidification rate (ECAR) was measured to assess the characteristics of the glycolysis process.

### Metabolic phenotyping using mitoplate analysis

MitoPlate S-1 plates from Biolog (Biolog, Hayward, CA, USA) were used according to manufacturer’s instructions. Each substrate was present in one well, and the mitochondrial substrate oxidizing capacity was measured by evaluating the rates of electron flow from the substrates to the electron transport chain, where a tetrazolium redox dye acts as an electron acceptor and turns purple after reduction. Briefly, leukemic cells were harvested from mono- or co-culture (as explained before), washed twice with 1x PBS and suspended in 1 mL of 1x Biolog Mitochondrial Assay Solution (BMAS). Per each well of the MitoPlate, 30 µL of cell suspension, 30 µL of reaction mix 15 µL BMAS, 10 µL Redox Dye MC, 2.5 µL saponin (75 µg/mL; SAE0073, Sigma-Aldrich, St. Louis, MO, USA) and 2.5 µL sterile water were added and incubated for 1 h at 37 °C. The MitoPlate was inserted in the Omnilog (Biolog, Hayward, CA, USA) set at 37 °C and the reduction of the tetrazolium redox dye MC was kinetically measured for 24 h, with 5 min intervals. The initial rate was calculated using the Biolog software data analysis (v. 1.7).

### CENCAT metabolic profiling

Human primary CD34 + cells were transferred onto the 96-well U-bottom plate (0.2 mln/well), followed by the original CENCAT protocol [19], which is a modification of the original SCENITH method [20]. Briefly, after 30 min of methionine depletion in methionine-free RPMI 1640 medium (Sigma Aldrich, #R7513) supplemented with 65 mg/L L-cystine dihydrochloride (MedChemExpress,#HY-W009203), 10% FBS, L-glutamine, cells were treated with metabolic inhibitors to shut down glycolysis (100 mM 2-deoxy-D-glucose; MedChemExpress, #HY-13966), mitochondrial metabolism (1 µM oligomycin A; MedChemExpress, #HY-16589) or both (combination of 2-deoxy-D-glucose and oligomycin A). Afterwards, cells were treated with a noncanonical amino acid analog of methionine - L-homopropargylglycine (HPG, 100µM; MedChemExpress, #HY-140345). After incubation and washing cells were stained with fixable viability dye (Invitrogen, #L34976), fixed and permeabilized. Finally, the cells were labelled through copper(I)-catalyzed azide-alkyne cycloaddition (CuAAC) in the CuAAC reaction mix (0.5 mM CuSO4, 10 mM sodium ascorbate, 2mM THPTA) with 0.5 µM AF647 Azide Plus (VectorLabs, #CCT-1482). The samples were analyzed on BD LSRFortessa cytometer, followed by data analysis using Diva and FlowJo software. Calculations of MFI values were made following the formula from the original protocol [19]: Glucose dependence (%): 100*((Ctr-DG)/(Ctr-DGO)); Mitochondrial dependence (%): 100*((Ctr-O)/(Ctr-DGO)); Glycolytic capacity (%): 100-(100*((Ctr-O)/(Ctr-DGO)); Non-glucose respiratory capacity (%): 100-(100*((Ctr-DG)/(Ctr-DGO)).

### Trans-SILAC MS

Cell labeling with heavy isotopologues of lysine and arginine was performed as previously described [5]. Briefly, the SILAC medium was supplemented with 10% of dialyzed FBS, 1% Pen/Strep, 0.274 mM L-lysine, and 1.15 mM L-arginine and filtered (0.22 μm pores). Donor cells were labeled with heavy isotopologues of lysine and arginine: L-lysine:2HCL (13C6, 99%; 15N2, 99%) and L-arginine: HCL (13C6, 99%; 15N4, 99%; Cambridge Isotope Laboratories). Cells were maintained for 9 days to enable complete labeling of the proteome. On day 8, donor cells were labeled with DiD dye as described above and on day 9 the co-culture was seeded as described above (Cell lines and co-culture system section).

### LC-MS/MS analysis

Mass spectrometry analysis was performed using an EASY nLC 1000 coupled to a Q-Exactive Plus mass spectrometer (ThermoFisher Scientific). Peptides were separated by a 180-min linear gradient of 95% solution A (0.1% formic acid in water) to 35% solution B (acetonitrile and 0.1% formic acid). Three washing runs preceded the measurement of each sample to avoid cross-contamination. The mass spectrometer was operated in the data-dependent MS-MS2 mode. Data was acquired in the m/z range of 300–1750 at a nominal resolution of 70,000.

Data were analyzed using the Max-Quant 1.5.3.12 platform, with the reference human proteome database from UniProt. False discovery rates of protein and peptide-spectrum matches (PSM) levels were estimated using the target-decoy approach at 0.01% (protein FDR) and 0.01% (PSM FDR), respectively. The minimal peptide length was set to 7 amino acids, and carbamidomethylation at cysteine residues was considered a fixed modification.

Oxidation (M) and Acetyl (Protein N-term) were included as variable modifications. Only proteins that were represented by at least two unique peptides in two biological replicates are shown and were further considered. The data analysis was performed using MaxQuant software and the MaxLFQ algorithm. Lists of proteins were analyzed using the Panther application for GeneOntology software, STRING-confidence view, and Venny 2.1 (http://bioinfogp.cnb.csic.es/tools/venny/index.html). Additionally, lists of proteins were grouped according to their molecular weights based on the UniProt database. The mass spectrometry data from this publication have been deposited to the ProteomeXchange Consortium via the PRIDE [https://www.ebi.ac.uk/pride] partner repository with the dataset identifier PXD013504.

### Metabolome MS

The normalized number of K562 cells (1.1 mln cells) were lysed with 100 µl of ice cold (−20 °C) methanol (LC–MS hyper grade, Merck). The samples were incubated in −20 °C for 2 h during which the samples were thoroughly vortexed every 20 min. After this time, the samples were centrifuged (30 min, 18,000 × g, −10 °C) and 80 µl of the supernatant was lyophilized with the use of a concentrator (Eppendorf). The lyophilizate was dissolved immediately before injection in a mixture of ddH2O and methanol (50:50 ratio) with addition of 0.1% formic acid, centrifuged (10 min, 18,000 × g, −10 °C) and 10 µl of sample was loaded for LC-MS. Untargeted metabolomics was analyzed using ultra-high-resolution Fourier-Transform Ion Cyclotron Resonance Mass Spectrometry with an electrospray ion source (ESI-FT-ICR-MS, SolariX 2xR 7 T, Bruker) in direct injection. The injection was made at a flow rate of 300 µl/h in both positive and negative polarity (with 3 technical replications of each sample). The ions accumulation time was set to 0.03 s., with the dry gas flow set to 4.0 L/min and drying temperature 200 °C. The capillary in the ion source was set to 3500 V (negative polarity) and 4500 V (positive polarity). The collected mass spectra (64 accumulated scans per spectrum) were analyzed using the T-Rex 2D algorithm (MRMS single spectra) in the MetaboScape 5.0 software (Bruker) and the identified compounds were assigned to specific signaling pathways using MetaboAnalyst 5.0 and the KEGG database.

### Total protein normalization

Cell pellets were solubilized in 200 µL of 0.5 M NaOH. For the total protein assay, the Bradford method was applied, using the premixed dye (Bio-Rad Protein Assay). The measurements were performed on 96-well plates using the microplate reader (Infinite M1000, Tecan, Switzerland). The protein amounts were calculated in reference to the standard curve prepared along with the samples based on the standard solution of bovine serum albumin (BSA).

### Analysis of existing gene expression data in CML patients with Imatinib resistance

Gene expression data from CML and healthy (control) CD34 + cells were obtained from Single-cell atlas of diagnostic Chronic Myeloid Leukemia bone marrow (scdbm) (http://scdbm.ddnetbio.com) based on Krishnan et al. A Single-cell Atlas Identifies Pretreatment Features of Primary Imatinib Resistance in Chronic Myeloid Leukemia [21]. A list of genes involved in the tricarboxylic acid cycle (GO:0006099) was obtained from the Gene Ontology database. Only data from CD34 + cells from the bone marrow were taken and presented here; CD34 + and CD34- cells were purified and subjected to scRNA-seq separately. CML patients were classified into three categories broadly based on recommendations by the European LeukemiaNet. Control – healthy participants (*n* = 8), A – imatinib good response (*n* = 9) – patients who either achieved major molecular response (MMR) after imatinib treatment within 12 months and/or deep molecular response (DMR); B – imatinib failure (*n* = 9) – patients who did not meet molecular and/or cytogenetic response benchmarks until 18 months of imatinib treatment but responded optimally to 2nd/3rd line TKI; C – pan-TKI resistance with eventual BC progression (*n* = 9) – patients who failed TKI therapy and progressed from chronic phase (CP) to the blast crisis (BC) stage of the disease.

### Statistical analysis

All of the experiments were performed in at least three independent biological repetitions. Trans-SILAC was done in two biological replicates. All of the data are presented as mean ± SD. Data were analyzed using GraphPad Prism (GraphPad Software, La Jolla, CA, USA). For Oroboros data analysis, unpaired parametric t-test was used. For further experiments, single comparisons were performed using ordinary one-way ANOVA test where normal distribution was checked with the Shapiro-Wilk test, and variance homogeneity with Brown-Forsythe test. For post hoc analysis Tukey test was performed. Two-way ANOVA performed in R was used to test the impact of imatinib and culture conditions on metabolic profiles. Values of *p* < 0.05 were considered statistically significant. *p < 0.05, **p < 0.01, ***p < 0.001, ****p < 0.0001.

## Results

### Direct contact with bone marrow stromal cells drives metabolic remodeling in leukemic cells

We previously showed that the bone marrow stroma mediates the protection of leukemic cells from drug-induced cell death, by upregulation of antiapoptotic proteins and signaling pathways [3–6]. This study verified the hypothesis that bone marrow stromal cells play a direct protective role in metabolic remodeling in leukemia, which correlates with attenuated apoptosis caused by imatinib.

**Fig. 1:**
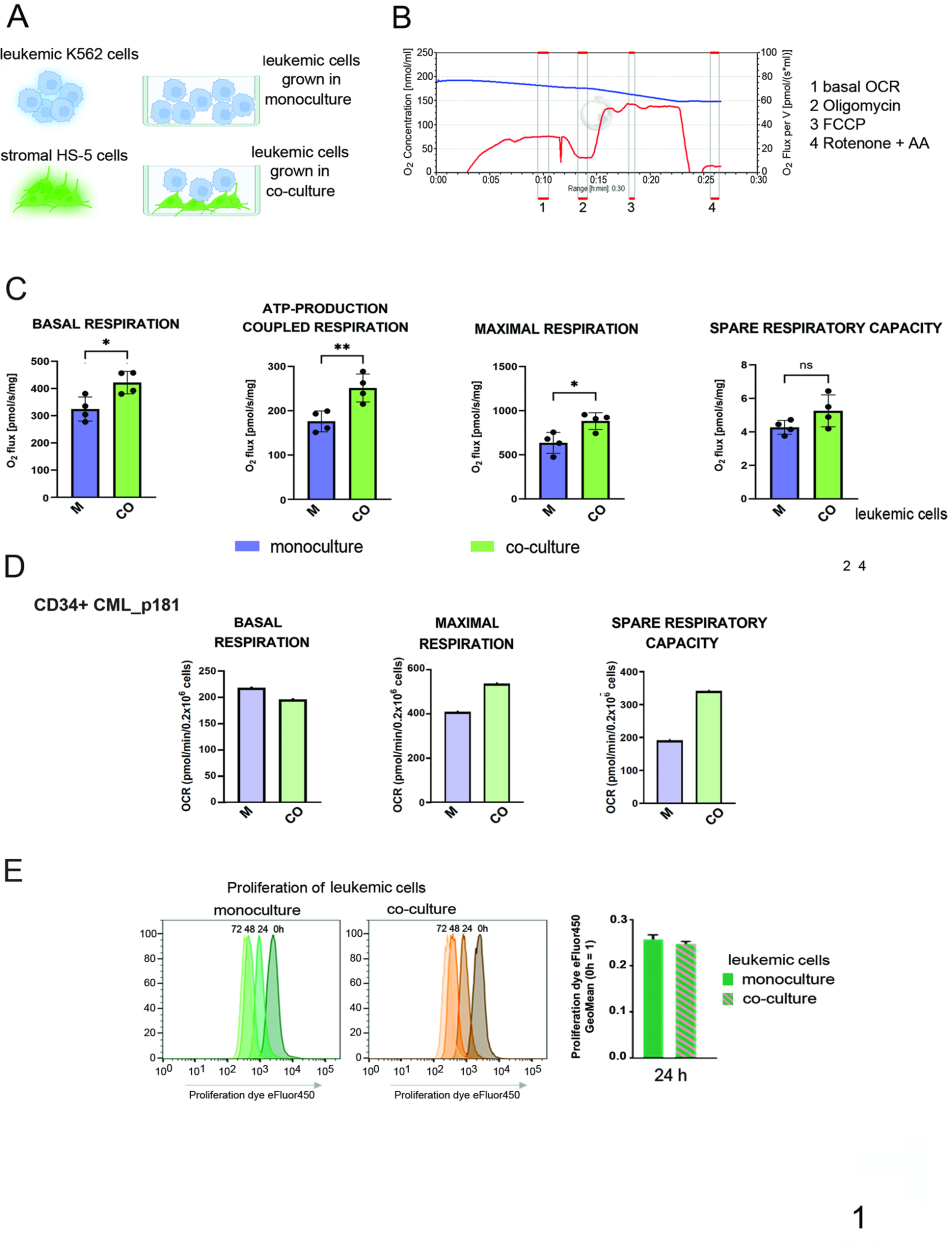
Co-culture with bone marrow stroma increases oxidative metabolism in leukemic cells.**A**. A scheme of the monoculture of K562 leukemic cells (M) or leukemia-stroma co-culture model (CO), in which HS-5 stromal cells were attached to the surface, and CML K562 cells were grown in suspension for 24–48 h. Experimental conditions are described in Material and Methods section. **B**. An exemplary oxygen consumption rate curve generated during Oroboros high-resolution respirometry. 1 - basal respiration, 2 - ATP synthase-independent respiration, 3 - maximal respiration, 4 - nonmitochondrial oxygen consumption. The red curve represents respiration rate, the blue curve depicts O2 concentration . Horizontal red lines in the upper part of each diagram represent timeframes of data collection. **C.** Metabolic profiles of leukemia cells grown in monoculture (M) or co-culture (CO). In co-culture conditions, K562 suspension cells were carefully harvested after gentle mixing the co-culture to detach leukemic cells from stroma (the contamination with floating stromal cells did not exceed 0.5%). Oxygen consumption rates are shown for basal respiration, ATP-production-coupled respiration, maximal respiration and spare respiratory capacity. Oxygen consumption is shown as O2 flux in pmol/s/mg. The data from *n* = 4 independent experiments are shown as mean ± SD. For statistical significance, co-culture conditions (CO) were compared to monoculture (M). An unpaired parametric t test was used, and p values < 0.05 were considered to indicate statistical significance. *p < 0.05, **p < 0.01, ***p < 0.001; ns = not significant. **D**. Metabolic profile of primary CML-CP CD34 + cells cultured alone (M) or in co-culture with HS-5 cells (CO). The effect of co-culture with HS-5 on CML-CP CD34 + cells’ basal respiration, maximal respiration and spare respiratory capacity measured with Seahorse technology. The data from *n* = 1 is shown. **E**. Left panel: representative flow cytometry histograms showing the proliferation of K562 cells cultured in monoculture or co-culture with HS-5 cells. Lower fluorescence intensity of Proliferation Dye eFluor450 corresponds to increased cell divisions. Right: K562 proliferation in mono- and co-culture shown as the ratio of geometric fluorescence intensity (GeoMean) of Proliferation Dye eFluor450 in K562 cells at 24 h to 0 h. The data from *n* = 3 independent experiments are shown as mean ± SEM

To obtain the bone marrow-like conditions, we opted for the co-culture model, which allows intercellular cross-talk and interactions between human erythroleukemia K562 or primary CD34 + CML cells growing in suspension and HS-5 stromal cells growing as an attached layer (Fig. 1A). As published, this model is used to mimic bone marrow-like conditions and has been successfully incorporated into our previous studies [3–6].

First, we assessed whether interactions with stromal cells remodel metabolic parameters of leukemic cells, by the oxygen consumption detected by respirometry method with glucose, pyruvate and glutamine as substrates (Fig. 1B-C; Supplementary Fig. 1 A). The general metabolic rate evaluated in leukemic cells after 24 h of growth either alone (monoculture - M) or in the presence of stromal layer (co-culture – CO) demonstrated that the leukemic cells co-cultured with stroma exhibited greater basal respiration, ATP-production coupled respiration and maximal respiration, compared with cells in the monoculture (Fig. 1C). The stroma-dependent increase was observed also for the spare respiratory capacity (Fig. 1C), which indicates the metabolic reserve which can be used under energetic needs like stress or drug treatment. The same tendency and stroma-dependent increase of maximal respiration and spare respiratory capacity was also found in primary CD34 + cells from CML patient, co-cultured with stromal cells (Fig. 1D), however more samples need to be tested.

Consistent with the increase in oxidative phosphorylation, a decrease in lactate production was observed (Supplementary Fig. 1B). Additionally, the glucose uptake by leukemic cells was not changed upon co-culture conditions (Supplementary Fig. 1C), indicated that the Warburg Effect is not upregulated in leukemia cells grown together with stroma compared to monoculture.

Assessment of the individual cell divisions using a proliferation dye and flow cytometry did not show any changes between mono- and co-culture (Fig. 1E), at least up to 72 h. Changes visible at 72 h resulted from high density and exceeded the experimental setup. The GeoMean values related to proliferation of leukemic cells grown for 24 h in both conditions (Fig. 1E, right graph) confirmed that metabolic changes observed in leukemic cells in co-culture were not associated with changes in proliferation.

To assess whether direct contact between stromal and leukemic cells is essential for metabolic remodeling, we physically separated both cell types using a trans-well co-culture system (Supplementary Fig. 1D). The trans-well system is a commonly accepted control for TNTs formation, which prevents direct interactions and the formation of intercellular connections like TNTs, however allowing for exchange of secreted factors. The physical separation inhibited the stroma-mediated remodeling of metabolic parameters in leukemic cells (Supplementary Fig. 1D, trans-well). This proved that physical cell-to-cell contact is critical and factors secreted by both cell types do not mediate the stroma-dependent metabolic remodeling in leukemia. Furthermore, no significant metabolic remodeling was observed when leukemic cells were cultured in conditioned medium (CM) derived from stromal cells cultured alone (Supplementary Fig. 1D, CM), excluding the role of factors secreted exclusively by stromal cells. This highlights the necessity of direct intercellular connections and a mechanism that is not related to exocrine secretion, for the stroma-mediated metabolic remodeling in leukemia.

Altogether, these findings suggest that stromal cells alter the metabolism of CML cells, in a direct cell-cell contact-dependent manner, promoting increased capacity of oxidative metabolism in leukemia.

### Stroma mediates metabolic plasticity in leukemic cells and protects from imatinib-induced metabolic decline and apoptosis

**Fig. 2:**
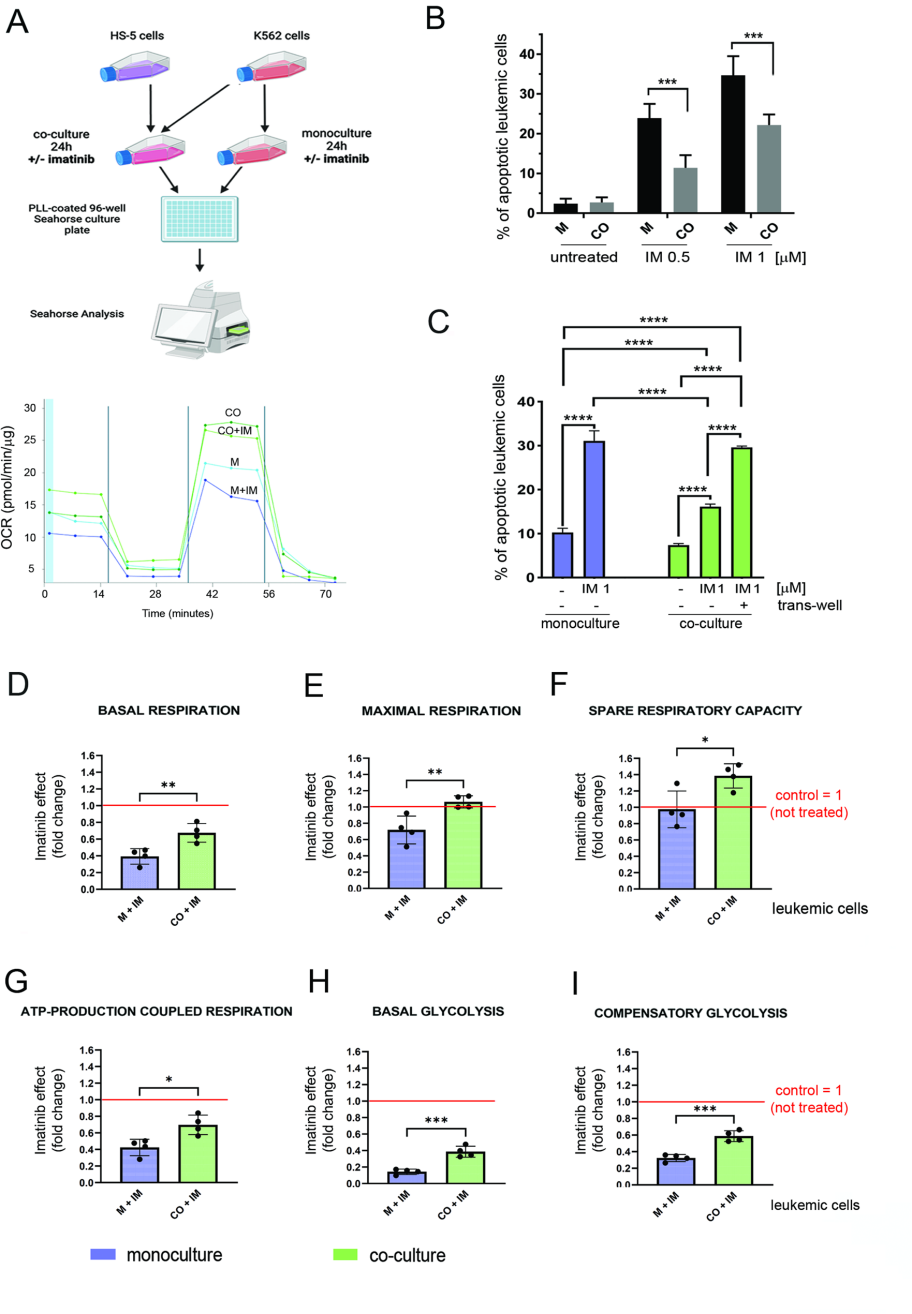
Stromal cells drive metabolic plasticity and protection from the harmful effect of imatinib in leukemic cells.**A** Left panel - scheme of the analysis of the metabolic profiles estimated by Seahorse Agilent in leukemia cells grown in monoculture (M) or co-culture (CO), untreated or treated with imatinib (+ IM). Right panel - A representative time-series graph showing changes of oxygen consumption rate (OCR) with time (pmol/min/µg) in leukemic cells grown under all conditions described above upon: basal respiration (untreated cells; time-points 1–3), inhibition of ATP-linked respiration (oligomycin treatment; time-points 4–6), maximal respiration (FCCP treatment; time-points 7–9) and non-mitochondrial respiration (antimycin A and rotenone treatment; time-points 10–12). **B** Apoptosis detected in leukemic cells grown in monoculture or co-culture, without treatment or treated with 0.5 or 1 µM imatinib (IM) for 48 h. Percentage of apoptotic leukemic cells was assessed by the Annexin V-7AAD cytometric assay, and Annexin V-positive cells were counted as apoptotic. The data from *n* = 3 are shown as mean ± SEM. For statistical analysis, co-culture conditions (CO) were compared to monoculture (M). Values of *p* < 0.05 were considered to indicate statistical significance. *p < 0.05, **p < 0.01, ***p < 0.001. **C** Apoptosis detected in leukemic cells grown in monoculture or co-culture, without treatment or treated with 1 µM imatinib (IM) for 48 h. To exclude the contribution of direct cell-to-cell contact between K562 and HS-5, a transwell system with 1 μm pore inserts was used. Percentage of apoptotic leukemic cells was assessed by the Annexin V-7AAD cytometric assay, and Annexin V-positive cells were counted as apoptotic. The data from *n* = 3 are shown as mean ± SD. Values of *p* < 0.05 were considered to indicate statistical significance. *p < 0.05, **p < 0.01, ***p < 0.001. **D-I.** The impact of imatinib on the metabolic profiles of leukemia cells grown in monoculture (M) or co-culture (CO), untreated or treated with imatinib (+ IM). In co-culture conditions, K562 suspension cells were carefully harvested after gentle mixing the co-culture to detach leukemic cells from stroma (the contamination with floating stromal cells did not exceed 0.5%). The effect of imatinib on the intensity of the indicated processes (**D-I**) is presented as relative abundances after normalization of OCR values to untreated conditions = 1 (red line), calculated for both conditions (monoculture and co-culture); basal respiration (**D**), maximal respiration (**E**), spare respiratory capacity (**F**), ATP-production coupled respiration (**G**), basal glycolysis (**H**) and compensatory glycolysis (**I**). The data from *n* = 4 independent experiments are shown as mean ± SD. For statistical significance, imatinib effects in co-culture (CO + IM) were compared to imatinib effects in monoculture (M + IM). Statistical analysis was performed using ordinary two-way ANOVA performed in R. Values of *p* < 0.05 were considered to indicate statistical significance. *p < 0.05, **p < 0.01, ***p < 0.001, ****p < 0.0001

To better understand the effect of stroma on metabolic response to drug treatment, we further assessed the bioenergetics of leukemic cells, growing either in monoculture or co-culture and treated with imatinib, a model tyrosine kinase inhibitor (TKI) and a first-line treatment for CML. For this we utilized the Agilent Seahorse system to maintain stable conditions during cell incubation, and calculated the effect of imatinib as a fold change normalized in respect to untreated cells = 1 (shown as red line), calculated separately for both conditions (monoculture and co-culture) (Fig. 2A and D-I).

Subsequently, apoptosis measured by AnnexinV/7AAD test in leukemic cells confirmed that co-culture protects leukemia from imatinib-induced cell death (Fig. 2B). Notably, this stroma-dependent protection from imatinib-induced apoptosis was not observed when the two cell types were physically separated using a trans-well (Fig. 2C), which as we already presented, protected also from metabolic remodeling (Supplementary Fig. 1D). This visibly demonstrated that the stroma-mediated resistance to imatinib depends on the direct physical interactions between both cell types.

Metabolic profiling of leukemic cells grown either in mono- or co-culture assessed that while treatment with imatinib greatly reduced both basal and maximal respiration, basal and compensatory glycolysis, and decreased ATP-production coupled respiration in leukemic cells, indicating the detrimental effects of imatinib on energy metabolism, the co-culture provided protection visible in all analyzed parameters (Fig. 2D-I). After co-culture with stroma, especially the reductions in oxidative phosphorylation (OXPHOS) and mitochondria-related parameters were substantially less pronounced (Fig. 2D-F). The maximal respiration in leukemic cells grown in co-cultures treated with imatinib was not only not reduced but was even elevated and greater than in untreated control (= 1, shown as red line) (Fig. 2E). In particular, the spare respiratory capacity which indicates the metabolic reserve, was significantly higher in leukemic cells co-cultured with stroma and treated with imatinib, and reached the fold of 1.4 respectively to untreated conditions (= 1, shown as red line) (Fig. 2F). Additionally, the stromal component attenuated the decrease in basal and compensatory glycolysis (Fig. 2H and I).

**Fig. 3:**
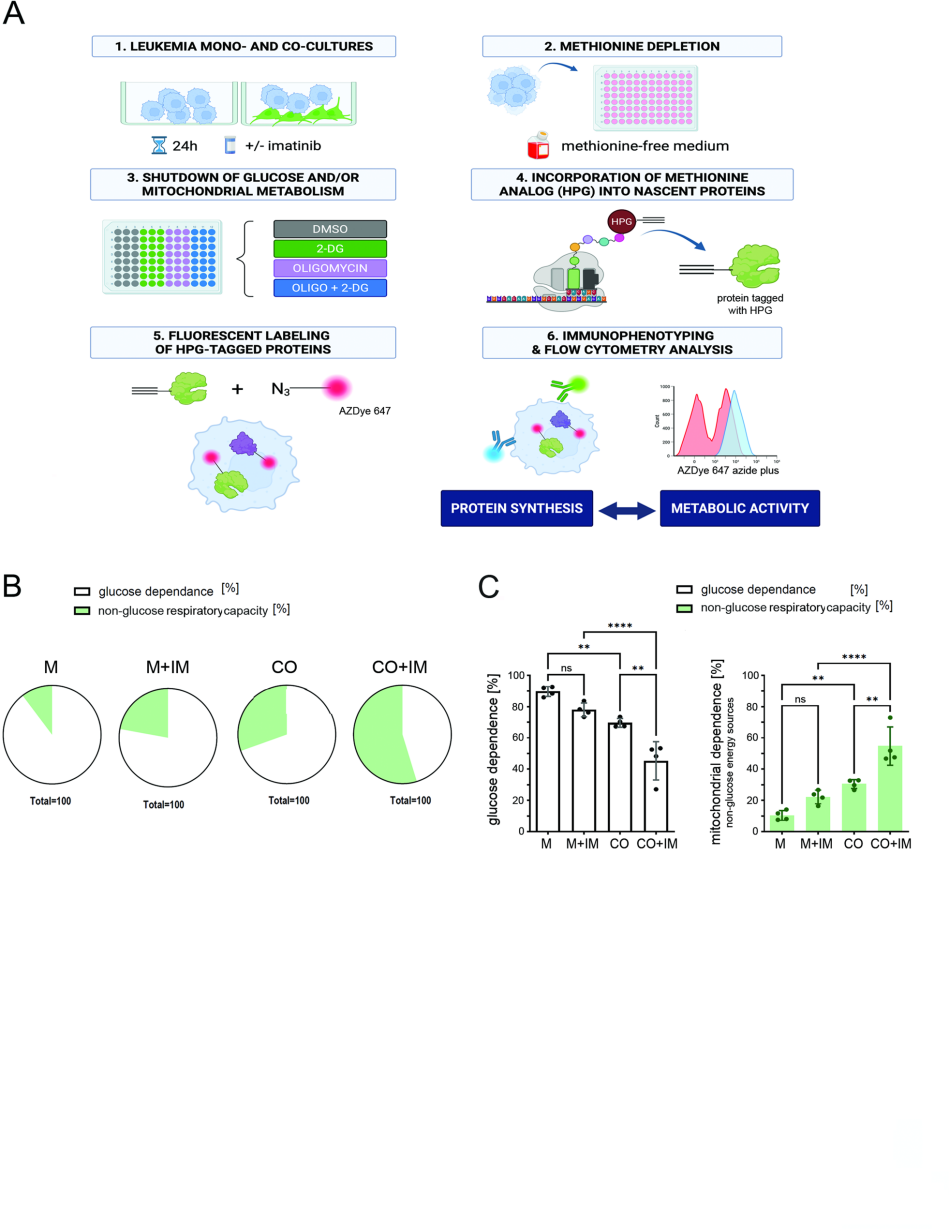
Stromal cells shift metabolism of leukemic cells towards non-glucose energy sources and promote fatty acid and amino acid oxidation in response to imatinib. **A** The scheme of CENCAT experiment. The metabolic profiles of cytometrically gated CML K562 cells, grown in monoculture or co-culture for 24 hours were assessed via the CENCAT method. The CENCAT uses flow cytometry to measure cellular metabolism based on changes in the level of translation in response to metabolic inhibitors. Cultures were either untreated or treated with imatinib for 24 hours, followed by treatment with metabolic inhibitors. Control cells (DMSO) or cells treated with oligomycin (O), 2-DG or the combination of both (DGO) were subjected to fluorescence detection via Azide-Az647. Calculations based on the Azide647-gMFI are shown in Supplementary Fig. 2A. **B **Pie charts representing the metabolic profile of glucose dependence versus non-glucose respiratory capacity measured by the CENCAT in K562 cells grown in monoculture (M) or co-culture with stroma (CO), untreated or treated with imatinib (IM). K562 cells were labeled with a fluorescent dye (eFluor450) before the co-culture for better separation. The gating strategy is shown in Supplementary Fig. 2B. **C **Statistical analysis of metabolic profiles of glucose dependence versus non-glucose respiratory capacity calculated based on the CENCAT method in leukemia cells grown in monoculture (M) or co-culture (CO), either untreated (B) or treated with imatinib (+IM). The data from n=4 independent experiments are shown as mean ± SD. For statistical significance, co-culture conditions (CO) were compared to monoculture (M). An unpaired parametric t-test was used, and p values <0.05 were considered to indicate statistical significance. *p < 0.05, **p < 0.01, ***p < 0.001, ****p<0.0001

The data we have presented so far have shown that the stromal component promotes preferential skewing of leukemic cells towards mitochondrial metabolism, together with no effect on glucose consumption. This is in line with the recent research which has highlighted the emerging role of non-glucose energy sources such as fatty acids and amino acids as a fuel in the mitochondrial metabolism in cancer [22, 23]. Moreover, high activity of non-glucose metabolism was associated with metabolic plasticity and resistance phenotype in leukemic cells [24–26]. To test the effects of stromal cells on non-glucose ATP sources of mitochondrial metabolism, we used the energetic metabolism profiling by the translation inhibition CENCAT (cellular energetics through non-canonical amino acid tagging) approach [19]. This flow cytometric method, based on the basic SHENITH principle that the rate of cellular protein synthesis is a proxy for metabolic activity and can be used to determine the cellular metabolic dependencies [20], allows to measure the ratio between glucose dependence and non-glucose respiratory capacity (Supplementary Fig. 2 A). To measure protein synthesis level detected upon selective blockade of metabolic pathways by 2-deoxyglucose, oligomycin or both together, CENCAT utilizes alkyne-bearing noncanonical amino acid tagging as a surrogate for ATP production and energetic metabolism. (Fig. 3A, Supplementary Fig. 2A, 2B).

We found that leukemia cells in the presence of stroma increased the non-glucose respiratory capacity (Fig. 3B and C). This was also consistent with previous observation that co-culture increased mitochondrial metabolism but without higher glucose consumption (Supplementary Fig. 1). Notably, when co-cultured cells were treated with imatinib, involvement of non-glucose capacity in leukemic cells further increased, suggesting the more efficient fuel of the activated respiratory metabolism (Fig. 3B and C). Finally, in leukemic cells grown in co-culture treated with imatinib, the non-glucose mitochondrial respiratory capacity represented over 50% (Fig. 3C). A similar trend was observed in patient’s CD34 + CML cells (Supplementary Fig. 3B, C), however more samples is needed to conclude. These data indicate a metabolic shift caused by the stromal microenvironment towards non-glucose mitochondria dependent metabolism.

Collectively, these findings support our previous observations and demonstrate that the bone marrow stromal cells profoundly alter metabolic plasticity of leukemic cells. Such reprogramming protects leukemic cells from metabolic drop and apoptosis after drug treatment, with greater capacity to produce energy based on non-glucose respiratory metabolism when co-cultured leukemic cells are treated with imatinib.

### The horizontal transfer of stromal intact mitochondria is not critical for stroma-mediated metabolic plasticity in leukemia

Having confirmed that direct intercellular connections are essential for the stroma-mediated metabolic plasticity in leukemia, we further investigated the role of direct cell-to-cell cargo transfer. One of direct cell-cell connections are tunneling nanotubes TNTs which are formed between distant stromal and leukemic cells [5]. Therefore, we hypothesized that there is a relationship between the direct horizontal transfer of cargo from the stroma to leukemia by TNTs and the stroma-dependent metabolic remodeling.

**Fig. 4:**
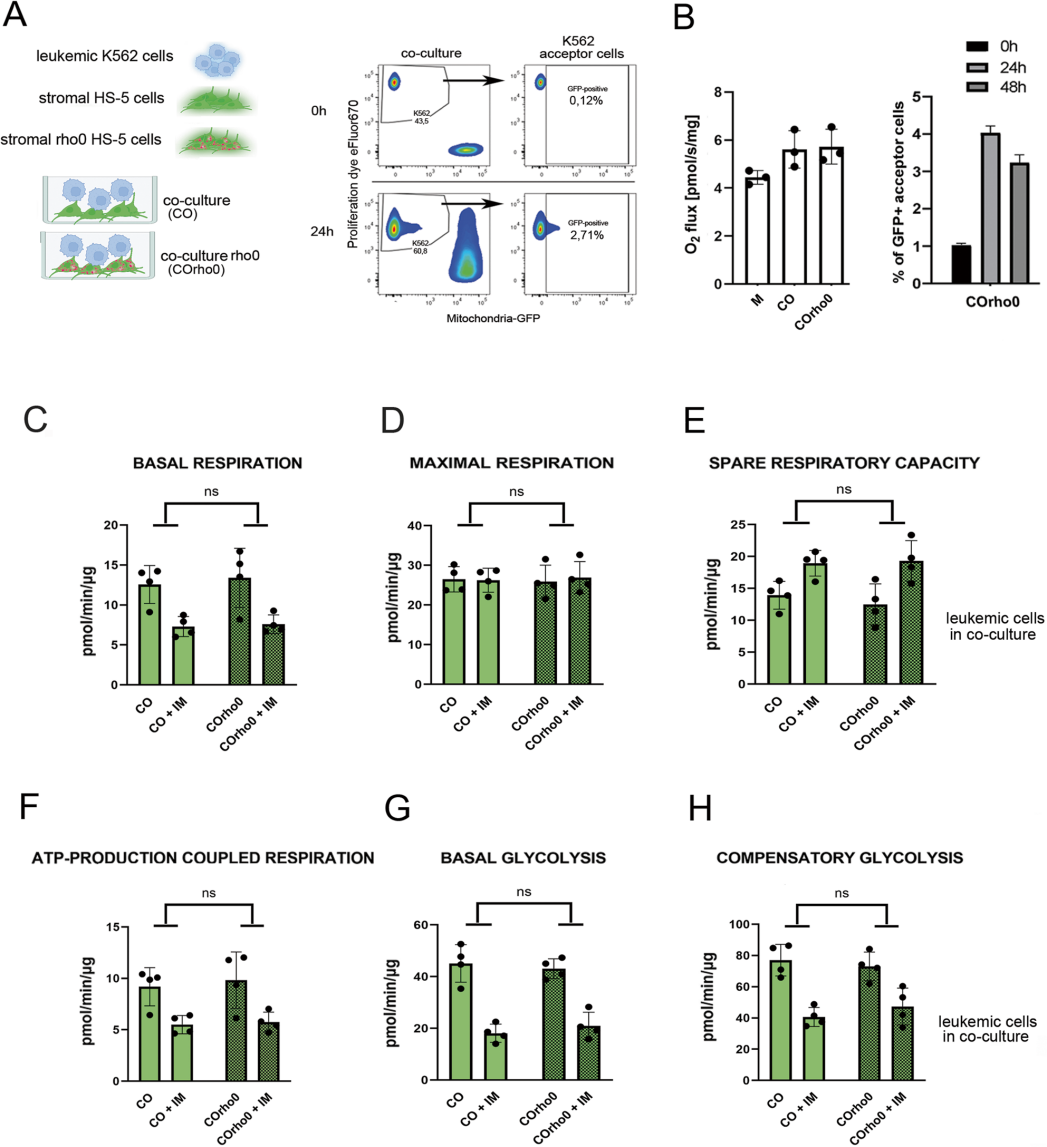
The horizontal transfer of stromal intact mitochondria is not critical for stroma-mediated metabolic plasticity in leukemia.**A** Left panel: the scheme of the monoculture of K562 leukemic cells (M) or two types of leukemia-stroma co-culture models where HS-5 cells contain GFP-positive mitochondria: with non-modified HS-5 stromal cells (CO) or with HS-5 rho0 stromal cells with defective mitochondrial metabolism (COrho0). Right panel: Representative dot plots showing the percentage of GFP-positive leukemia recipient acceptor cells (K562 acceptor cells) under control conditions and after 24 h of co-culture. In “co-culture” dot plots, both cell types (K562 and HS-5) are presented. The time point “0” shows cytometric separation of GFP-positive HS-5 donors from eFluor670-positive K562 cells in co-culture sample. The 24 h dot plots present the GFP-mitochondria uptake by K562 acceptor recipient cells. The “K562 acceptor cells” dot plots show only leukemic recipients gated on eFluor670 + population. **B** Left panel: oxygen consumption rates measured in leukemic cells grown in monoculture (M), co-culture (CO) or co-culture with stromal rho0 cells with defective mitochondrial metabolism (COrho0). In co-culture conditions, K562 suspension cells were carefully harvested after gentle mixing the co-culture to detach leukemic cells from stroma (the contamination with floating stromal cells did not exceed 0.5%). Data from *n* = 3 independent experiments are shown as mean ± SD. Right panel: Transfer of mitochondria estimated by flow cytometry in co-culture of HS-5 stromal cells expressing GFP-tagged mitochondria with fluorescently labeled with proliferation dye eFluor670 leukemic cells. The percentage of GFP-positive leukemic cells (GFP + acceptor recipients) in co-culture with stromal HS-5 rho0 cells with defective mitochondrial metabolism after 24 h and 48 h of co-culture. Data from *n* = 4 independent experiments are shown as mean ± SD. **C-H** The metabolic profiles assessed by the Seahorse technology in leukemia cells grown in non-treated co-cultures or imatinib-treated (+ IM) co-cultures of leukemia cells with either metabolically healthy HS-5 cells (CO) or with stromal rho0 cells with defective mitochondrial metabolism (COrho0). In co-culture conditions, K562 suspension cells were carefully harvested after gentle mixing the co-culture to detach leukemic cells from stroma (the contamination with floating stromal cells did not exceed 0.5%). The oxygen consumption rates are shown for basal respiration (**C**), maximal respiration (**D**), spare respiratory capacity (**E**), ATP-production coupled respiration (**F**), basal glycolysis (**G**) and compensatory glycolysis (**H**). Data from *n* = 4 independent experiments are shown as mean ± SD. Statistical significance in C-H panels tested by comparison of indicated pairs. Unpaired parametric t-test was used, p-values < 0.05 were considered as statistically significant; *p < 0.05, **p < 0.01, ***p < 0.001, ****p < 0.0001

To investigate the possible mechanism, first we examined the transfer of metabolically active mitochondria from stromal to leukemic cells. Genetically modified HS-5 cells containing GFP-positive mitochondria, which were sorted to obtain a population with minimum 98.9% of positivity were applied to accurately quantify the transfer, and to avoid false positives due to nonspecific staining with fluorescent mitochondria-tracking agents (Fig. 4A, Supplementary Fig. 4A). Leukemic cells were additionally fluorescently tracked with proliferation dye eFluor670 (Fig. 4A, left panel; co-culture, 0 h). After 24 h of co-culture, approximately only 3.5% of leukemic recipients had received GFP-positive stromal mitochondria and were detected as eFluor450 + GFP+ (Fig. 4A; Supplementary Fig. 4A). The use of trans-well for co-culture of leukemic cells with mito-GFP stromal cells or culturing leukemic cells with stromal conditioned medium (CM) prevented mitochondrial transfer, confirming the necessity of direct contact and excluding the role of secreted factors (Supplementary Fig. 4A). Our previously published data showed that imatinib did not increase mitochondrial transfer during the first 24 h of treatment, when metabolic remodeling occurred [5]. Additionally, co-culture did not lead to significant changes in the levels of the respiratory chain subunits (Supplementary Fig. 4B) or in the mitochondrial mass measured in leukemic cells (Supplementary Fig. 4E).

This data indicated that mitochondria are not efficiently directly transferred from stroma to leukemia cells either in control or under imatinib treatment conditions.

Apart from the direct transfer, we next assessed whether the active state and oxidative phosphorylation of stromal mitochondria in general may contribute to metabolic protection. To verify this, the rho0 stromal cells (Supplementary Fig. 4 C), which are characterized by depletion of mitochondrial DNA, dysfunctional respiratory chain and impaired OXPHOS metabolism were applied. Visualization of mitochondria expressing GFP protein confirmed disrupted mitochondrial network in rho0 stromal cells (Supplementary Fig. 4D).

Co-culture of leukemic cells with HS-5-rho0 cells did not impact either the respiratory capacity (Fig. 4B, left graph) or the direct transfer of mitochondria to leukemic cells (Fig. 4B, right graph). Furthermore, metabolic profiling showed that co-cultures with rho0 stromal cells did not alter the effects of stroma on metabolic parameters in imatinib-treated leukemia cells, compared to co-cultures with healthy (rho+) HS-5 cells (Fig. 4C and H), suggesting the involvement of a different mechanism. Importantly, protection from the metabolic drop after imatinib treatment (especially visible in maximal respiration (Fig. 4D) and spare respiratory capacity (Fig. 4E) was equally effective in co-culture with rho0 stroma as in co-culture with stroma with fully active mitochondria.

Altogether, we therefore conclude that the horizontal transfer of stromal mitochondria with functional oxidative phosphorylation chain is not essential for the stroma-mediated metabolic plasticity induced in leukemic cells, further supporting a different possible mechanism.

### Stromal cells directly transfer TCA-related proteins to leukemia recipients

Our previous study demonstrated that membrane vesicles together with proteins can be transferred by TNTs from stroma to leukemia [5]. Therefore, as another mechanism possibly involved in the stroma-driven metabolic remodeling, we assessed the transfer of metabolic proteins along with stromal membrane vesicles.

**Fig. 5:**
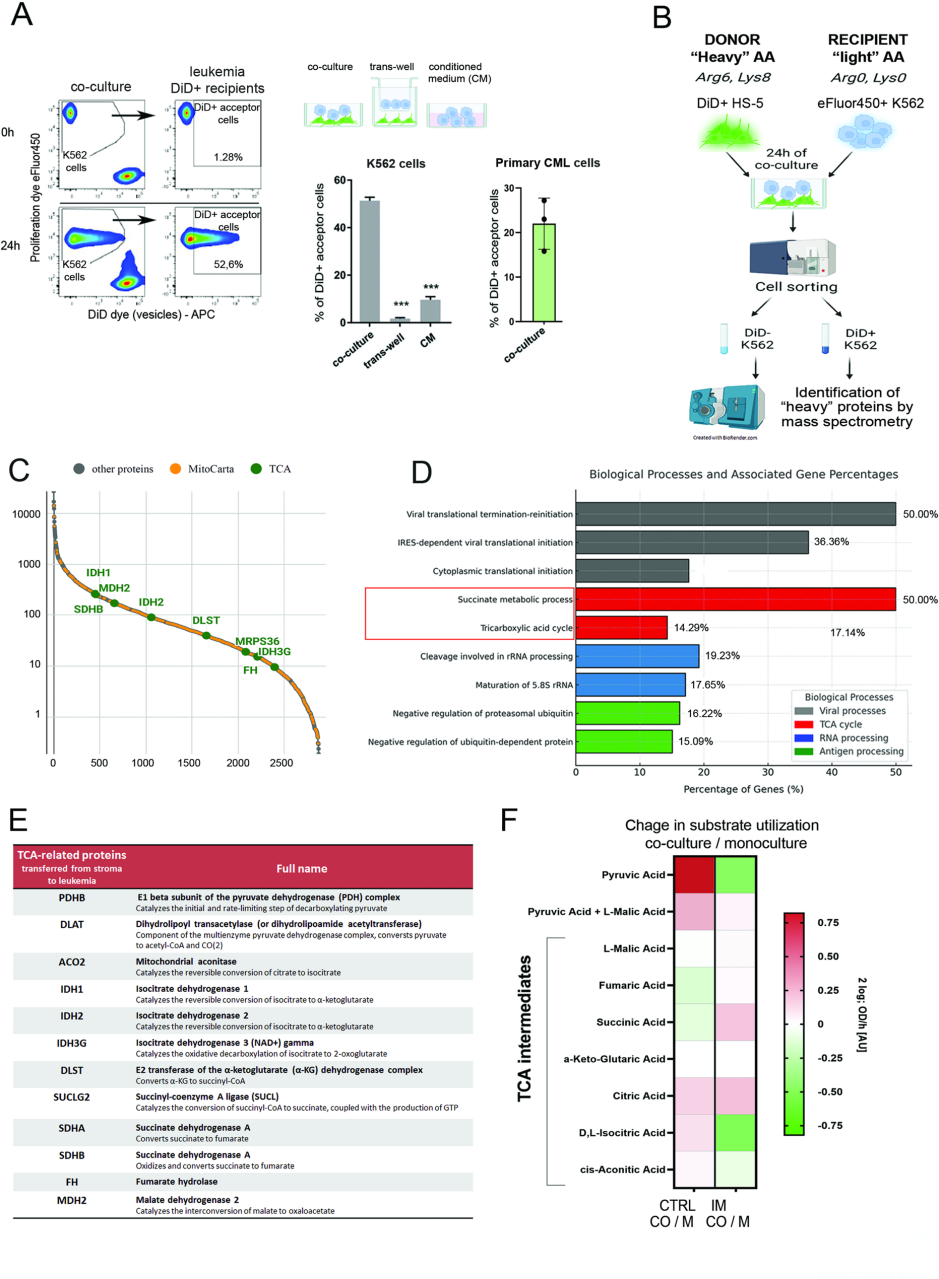
**A** Membrane vesicle transfer from stromal to leukemic cells is efficient in leukemia and requires direct cell-to-cell contact. Transfer of DiD+ vesicles estimated by flow cytometry in co-culture of HS-5 stromal cells stained with DiD dye to track membrane vesicles, with leukemic acceptor recipient cells tracked with the proliferation dye eFluor450 (K562 acceptor cells) for flow cytometric separation. Left panel- Representative dot plots showing the percentage of DiD-positive (DiD+) leukemia acceptor recipient cells growing in co-culture for 24 h. In “co-culture” dot plots, both cell types (K562 and HS-5) are presented. The time point “0” shows cytometric separation of APC-positive HS-5 donors from eFluor450-positive K562 cells in co-culture sample. The 24 hours dot plots present the DiD-positive vesicles uptake by K562 acceptor recipient cells. The “K562 acceptor cells” dot plots show only leukemic recipients gated on eFluor450+ K562 population as shown. Right panel - the percentage of DiD-positive K562 recipients (DiD+ acceptor cells) in co-culture with cell‒cell contact media, in the trans-well setup or when leukemic cells were grown in stromal conditioned medium (CM). The percentage of DiD-positive primary CML cells (DiD+ acceptor cells) in direct co-culture with HS-5 cells. CML cells were identified based on CD45+ phanotype. The data from n=3 are shown as mean ± SD. Statistical significance tested by comparison of the results for trans-well and CM conditions, with the results for co-culture. An unpaired parametric t test was used, and p values <0.05 were considered to indicate statistical significance. *p < 0.05, **p < 0.01, ***p < 0.001. **B** Scheme of the MS proteomics trans-SILAC experiment. HS-5 cells were incubated 9 days with heavy AA and stained with DiD+ to stain vesicles. Separately, K562 cells were incubated 9 days with light AA and tracked with eFluor 450 proliferation dye for improved separation. Then both cell types were mixed 1:1 and co-cultured for 48 hours. K562 eFluor450+ DiD+ or K562 eFluor450+ DiD- recipient cells were gated and sorted, followed by trans-SILAC MS. **C** Abundance plot illustrating the relationship between the sum of identified heavy-labeled proteins in DiD+ leukemic cells (Y-axis) noted in MitoCarta (orange) and individual proteins ranked along the X-axis. Heavy proteins overrepresented in DiD+ versus DiD- group, involved in the TCA cycle are highlighted in green. **D** Chart of the gene annotation of biological processes (Gene Ontology BP) associated with identified heavy-labeled proteins. Percentage values are calculated relative to the total number of heavy-labeled proteins identified in DiD-positive leukemic recipients. Proteins annotated with the TCA cycle are shown in a red frame. **E** The list of TCA-related proteins transferred from stroma to leukemia. **F** The oxidation of metabolic substrates related to TCA cycle activity measured with Mito Plate assay in K562 cells growing in monocultures (M) or co-cultures with HS-5 (CO), treated (IM) and untreated (CTRL) with 1 µM imatinib. Data are presented as logarithmic fold change in the CO/M ratio heatmap.

For this we applied a co-culture model in which stromal cells (vesicle donors) were fluorescently stained with DiD dye to visualize membrane vesicles and to enable detection of fluorescent stromal vesicles transferred to recipient leukemic cells by flow cytometry. Leukemic cells were fluorescently stained with proliferation dye eFluor450 (Fig. 5A). We observed that after 24 h co-culture, approximately 50% of the leukemic K562 cells and 25% of primary CML cells were DiD-positive, therefore represented the DiD stained stromal vesicle recipients (Fig. 5A, Supplementary Fig. 3A). Stromal vesicles uptake was not diminished by imatinib treatment (Supplementary Fig. 3A). The vesicle transfer was significantly reduced when cells were co-cultured in trans-well or CM conditions (Fig. 5A, left graph), confirming the necessity of direct interactions. This data were consistent with our previous findings on metabolic protection, which altogether suggested that direct transfer of membrane vesicles is necessary to obtain stroma-mediated protective effects.

This data demonstrate that membrane vesicles are transferred from stromal to leukemic cells. This vesicle transfer needs the direct cell-cell contact and therefore is associated with previously observed metabolic remodeling and protection from imatinib.

Consequently, we hypothesized that the direct horizontal transfer of proteins together with vesicles might be responsible for the metabolic rearrangement. To identify proteins directly transferred from stroma to leukemia, we applied previously described [5] trans-SILAC mass spectrometry (MS) proteomics combined with fluorescent tracking of both cell types and stromal vesicles to enable separation and sorting by flow cytometry (Fig. 5B). This stroma-leukemia model mimicked the physiological conditions, when leukemic cells residue in the BMM. After 24 h of co-culture, leukemic cells that received fluorescent vesicles from stroma (eFluor450 + DiD + K562 population) were sorted, and the stromal proteins labeled with “heavy” isotopes that were transferred along with membrane vesicles (the DiD + fraction) to leukemia cells in co-culture were identified by mass spectrometry (Fig. 5B – experimental setup).

In total, we identified 3821 “heavy” peptides in the DiD + leukemic recipients, including 918 annotated peptides of mitochondrial origin (24%). Among the overrepresented proteins, 9 were particularly highly enriched and annotated with the tricarboxylic acid (TCA/Krebs) cycle based on the MitoCarta database. The abundance plot (Fig. 5C) illustrates the relationship between the cumulative events of the identified heavy-labeled proteins in the DiD + sample and the individual proteins, ranked along the X-axis, with TCA cycle proteins highlighted in green. The gene annotation of biological processes (Gene Ontology BP) revealed that co-culture resulted in the transfer of various functional groups of proteins. Notably, twelve highly overrepresented proteins, referred to as “heavy” (what means that they are of stromal origin), were associated with the aerobic metabolism and TCA cycle (annotated as “succinate metabolic processes” (50%) and “tricarboxylic acid cycle” (14.29%) (Fig. 5D). These included the SUCLG2, SDHA, SDHB, DLAT, PDHB, IDH1, IDH2, IDH3G, DLST, FH, ACO2 and MDH2 proteins, all involved in the TCA-related metabolic aerobic processes, either directly or by fuel of the TCA cycle (Fig. 5E).

Importantly, the two identified “heavy” TCA proteins, dihydrolipoamide S-succinyltransferase (DLST) and aconitase 2 (ACO2), were identified together with a unique cleavable peptide localization sequence necessary for targeting mitochondria. This finding suggested that the proteins can be intercellularly transferred into the mitochondria in the form of precursor proteins, however we cannot exclude that also other proteins (not detected in this experimental setup) also contained such peptide localization domain. To directly visualize and proof the transfer of identified proteins, we selected DLST, a key TCA regulator linked to metabolic plasticity in cancer [27], as an example. HS-5 cells were genetically engineered to express the fluorescent DLST-GFP protein (Supplementary Fig. 5 A, 5B), sorted and used for co-culture with K562 cells additionally tracked with fluorescent dye eFluor450 (Supplementary Fig. 5B). In co-culture, the DLST-GFP-positive leukemic recipient cells were detected already at 6 h and the percentage of GFP-positive leukemic cells further increased at 24 h, and imatinib did not significantly decrease this transfer (Supplementary Fig. 5 C). These results provide clear evidence that stromal proteins identified by trans-SILAC like DLST, can be successfully transferred to leukemic cells and that this transfer is not impaired by imatinib treatment. Nevertheless, we do not point this particular protein as a unique driver, rather the whole group of metabolic proteins transferred from stroma mediates the protective phenotype. Altogether this data confirmed that stromal cells transfer TCA-related proteins to leukemia recipients and such transfer still occurs when leukemic cells are treated with imatinib.

To better characterize the functional effects of the observed transfer of TCA proteins and assess whether this transfer correlates with increased TCA cycle activity, we used cell-based phenotyping assay based on MitoPlate technology and measured the oxidation of metabolic substrates related to TCA cycle (Fig. 5F). This was assessed on leukemic cells grown in monoculture or co-culture (BMM), untreated or treated with imatinib, to mimic the drug treatment conditions. This allowed to verify whether the stroma-driven transfer improves activity of TCA cycle and whether this increased activity is still present when leukemic cells are treated with imatinib.

K562 cells after co-culture showed an increased capacity to oxidize TCA substrates such as pyruvic acid, malic acid, citric acid, isocitric acid and aconitic acid compared to monoculture, as seen by the logarithmic fold change in the CO/M ratio heatmap (Fig. 5F, CTRL). Notably, consumption of almost all TCA intermediates was also enhanced in co-cultures leukemic cells versus monocultured (Fig. 5F, IM). This indicated higher dynamics and activity of TCA cycle in leukemic cells treated with imatinib, when growing in the presence of stroma. However, the drop in pyruvic acid and isocitric acid consumption might indicate the change in TCA cycle dynamics and the change in type of TCA fuel.

### Stroma-mediated metabolome remodeling activates the TCA cycle-related aerobic metabolism and protects from metabolic decline and oxidative stress after imatinib

**Fig. 6:**
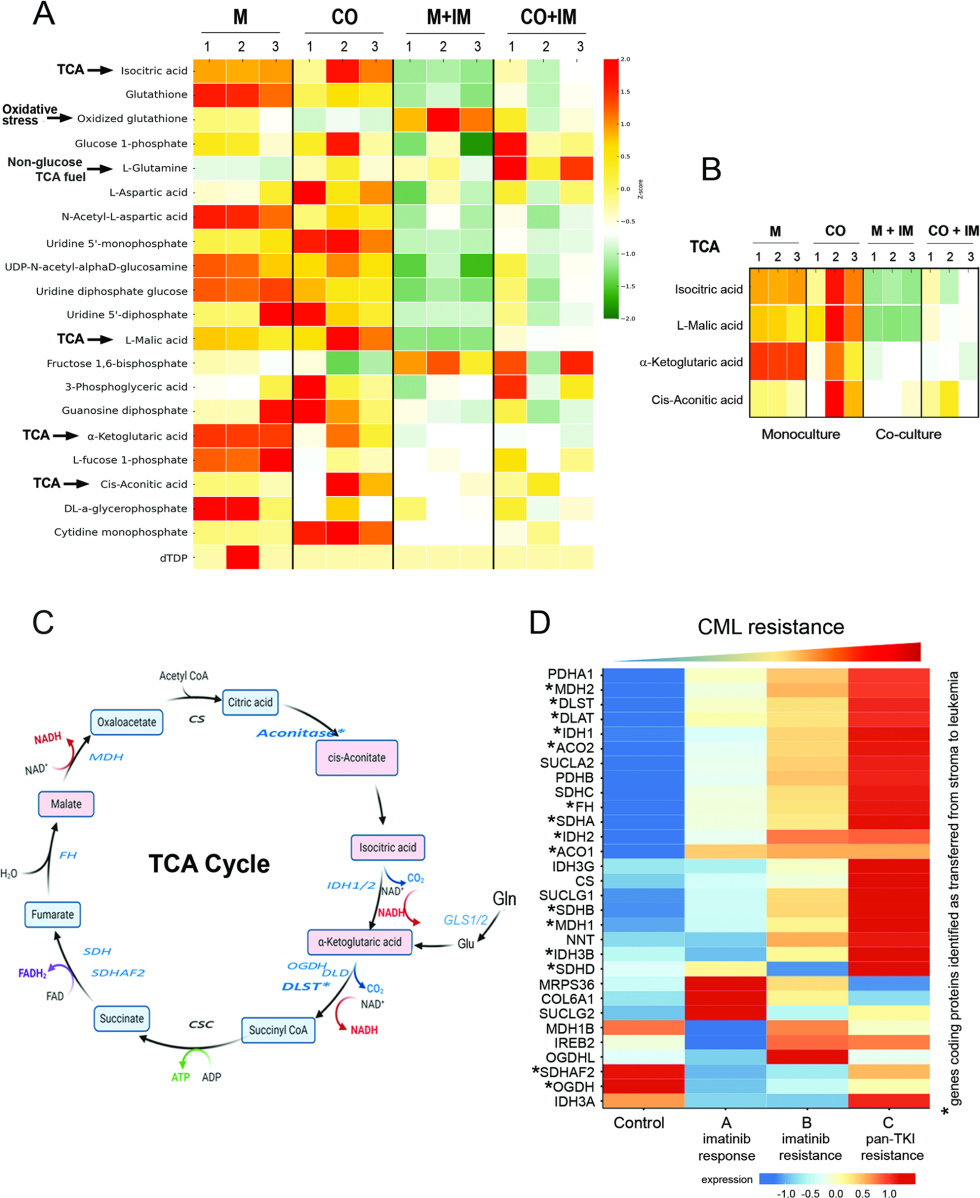
Stroma-dependent metabolome remodeling in leukemic cells. **A** Heatmap displaying the top 20 most significantly altered metabolites in leukemic cells according to the following experimental conditions: M (monoculture), CO (co-culture of stromal and leukemic cells), M + IM (monoculture with imatinib), and CO + IM (co-culture with imatinib), identified through LC‒MS analysis. Heatmap displaying the top 20 most significantly altered metabolites in leukemic cells according to the following experimental conditions: M (monoculture), M + IM (monoculture with imatinib), CO (co-culture of stromal and leukemic cells), and CO + IM (co-culture with imatinib), identified through LC‒MS analysis. Metabolome data analysis was performed based on three independent experiments (numbered: 1, 2, 3 on the heatmap) [–]. Metabolite levels are presented as z scores of signal intensities. **B **A subset of four overrepresented differentially abundant metabolites from the tricarboxylic acid (TCA) cycle identified among the top 20 altered metabolites is highlighted. **C** A schematic representation of the effects of co-culture on the TCA cycle. The blue font and blue frame indicate the proteins identified as being transferred from the stroma to the leukemia stroma. Frames with pink backgrounds indicate metabolites altered by co-culture. **D** Heat map displaying the expression of genes involved in the regulation of TCA cycle analyzed in CD34 + cells based on data from Single-cell atlas of diagnostic Chronic Myeloid Leukemia bone marrow dataset (http://scdbm.ddnetbio.com). CD34 + and CD34- cells were purified and subjected to scRNA-seq separately. CML patients were classified into three categories broadly based on recommendations by the European LeukemiaNet. Control – healthy participants (*n* = 8), A – imatinib good response (*n* = 9) – patients who either achieved major molecular response (MMR) after imatinib treatment within 12 months and/or deep molecular response (DMR); B – imatinib failure (*n* = 9) – patients who did not meet molecular and/or cytogenetic response benchmarks until 18 months of imatinib treatment but responded optimally to 2nd/3rd line TKI; C – pan-TKI resistance with eventual BC progression (*n* = 9) – patients who failed TKI therapy and progressed from chronic phase (CP) to the blast crisis (BC) stage of the disease. Proteins which were identified in our study as transferred from stromal cells to leukemic cells are marked with asterisk

So far, our data demonstrated that stromal TCA-related proteins are directly transferred to leukemic cells and this is associated with increased TCA cycle dynamics and activity also after imatinib treatment under co-culture conditions, indicating the stroma-dependent protection from metabolic decline. Next, to provide functional evidence for the effect of stroma on metabolite composition in leukemia cells, we applied the LS-MS-based metabolomics analysis (Fig. 6A, B). Metabolome profiling of the top 20 most common metabolites revealed that stroma-mediated protection was associated with increased levels of TCA-related metabolites such as isocitric acid, L-malic acid, ketoglutaric acid and cis-aconitic acid (Fig. 6A and B). Imatinib treatment of leukemia cells grown in monoculture decreased the levels of nearly all the identified metabolites, but increased oxidized glutathione, altogether indicating a metabolic decline, nutrient deprivation and high oxidative stress. The presence of BM stroma mitigated the effects of imatinib, and protected from metabolic drop. This was visible as higher levels of TCA-related metabolites in co-culture (CO + IM) compared to monoculture treated with imatinib (M + IM), confirming the stroma-dependent protection from metabolic decline after drug treatment. Elevated levels of TCA metabolites in co-culture, in comparison with imatinib-treated monoculture, were consistent with the proteome and MitoPlate mitochondrial phenotype analyses.

Remarkably, increased levels of L-Glutamine (Gln), a non-glucose energy source and efficient TCA cycle fuel to replenish metabolic intermediates under higher needs [28] were also observed in co-culture conditions, especially after imatinib treatment (Fig. 6A). Gln is required for the biosynthesis of various molecules and energy production in TCA and is the most abundant amino acid in plasma consumed by tumor cells at much greater rates that any other amino acids [29]. This observation was consistent with previous data which showed a stroma-driven remodeling towards a non-glucose dependent respiratory metabolism in leukemic cells, strongly visible when leukemic cells grown in the BMM were treated with imatinib. In parallel, analysis of the trans-SILAC MS proteomics identified increased levels of stromal mitochondrial Gln transporters from SLC25 family in leukemic cells.

Moreover, the metabolome analysis indicated that the presence of stromal cells reduced oxidative stress (lower levels of oxidized glutathione). This was associated with decreased ROS levels measured by flow cytometry (Supplementary Fig. 6A) and strongly induced expression of SOD2 in leukemic cells caused by the stromal component, either without or with imatinib (Supplementary Fig. 6B), which are necessary to prevent oxidative stress in response to increased TCA cycle and to imatinib treatment.

Altogether these data indicate that the stroma-mediated metabolic protection activates the TCA cycle activity with Gln input as TCA fuel, together with simultaneous protection from oxidative stress.

Figure 6C illustrates the components of the TCA cycle-related metabolism activated in leukemic cells by the presence of BM stroma. These findings are largely consistent with predictions and highlight the stroma-driven metabolic remodeling of leukemia cells towards the OXPHOS and TCA activity, corresponding with protection from imatinib effects.

### Higher expression of TCA-related genes in CML CD34 + cells corresponds to TKI resistance

To better define the possible role of observed changes in CML therapy, we adopted an open data base from Single-cell atlas of diagnostic Chronic Myeloid Leukemia bone marrow (scdbm) (http://scdbm.ddnetbio.com) [21] to check whether expression of TCA-related genes, particularly these encoding the proteins identified by us as transferred from stroma to leukemia, correlate with imatinib and new generation TKI resistance in CML patients. Data from single-cell RNA-seq profiling of bone marrow CD34 + cells isolated from either healthy donors (control) (*n* = 8) or CML patients classified based on European LeukemiaNet into three groups (described in detail in the Methods section): (A) imatinib good response (*n* = 9); (B) imatinib resistance (*n* = 9) and (C) pan-TKI resistance with progression into blast crisis (BC) (*n* = 9) were used. Then, we re-analyzed these data according to genes annotated as tricarboxylic acid cycle (GO:0006099) by the Gene Ontology database (Fig. 6D). Gene expression profiles (with genes encoding proteins that we identified as being transferred from stroma to leukemia cells or increased in metabolome of leukemia cells in a stroma-dependent manner marked with an asterisk) presented as a heatmap revealed significant correlation between the higher levels of expression of TCA components and resistance to imatinib and newer TKIs (Fig. 6D). In healthy donors expression of nearly all genes of our interest was very low and only slightly increased in imatinib good responders (group A). The levels of TCA-related genes significantly increased in CD34 + bone marrow CML cells from patients with resistance to imatinib (group B). Importantly, expression of TCA-related genes, including genes marked with an asterisk (transferred or activated by the BMM) further increased in CD34 + CML cells resistant to either imatinib or 2nd/3rd generation TKIs (group C, pan-TKI resistance). This indicates that increased levels of TCA-related genes correlate with resistance not only to imatinib but also to next generation TKIs, indicating a general clinical importance.

Altogether, these data showed that enhanced TCA-related genes expression correlates with a resistant phenotype and clinical failure after treatment with TKIs - either imatinib or new generation TKI. This also pinpoints these proteins as important players in the development the of BMM-dependent resistance in CML and interesting targets for therapeutic intervention.

### Targeting the TCA-related metabolism overcomes the stroma-mediated protection from imatinib

Taken together, our data obtained so far suggest that the stroma-mediated increase of the TCA cycle-related metabolism with Gln input is associated with stroma-mediated protection from imatinib. To validate the proposed role of the identified elements of the stroma-mediated protection, and verify the clinical potential of our findings, we applied telaglenastat (CB-839). Telaglenastat is inhibitor of glutaminase 1 (GLS1), which emerges as an essential non-glucose source and TCA fuel to refill TCA cycle intermediates, especially in cancer cells under needs or stress [30]. As a result, telaglenastat inhibits TCA cycle function by limiting the supply of important TCA precursor and inhibited TCA fuel even in the presence of TCA enzymes. Importantly, our proteomics and metabolomics study showed increased stroma-dependent activity of TCA and glutamine upregulation especially under imatinib treatment, providing a solid basis for the use of this treatment. Currently telaglenastat is in phase I/II in solid tumors [31] and as part of the combined therapy in myelodysplastic syndrome [32, 33].

**Fig. 7:**
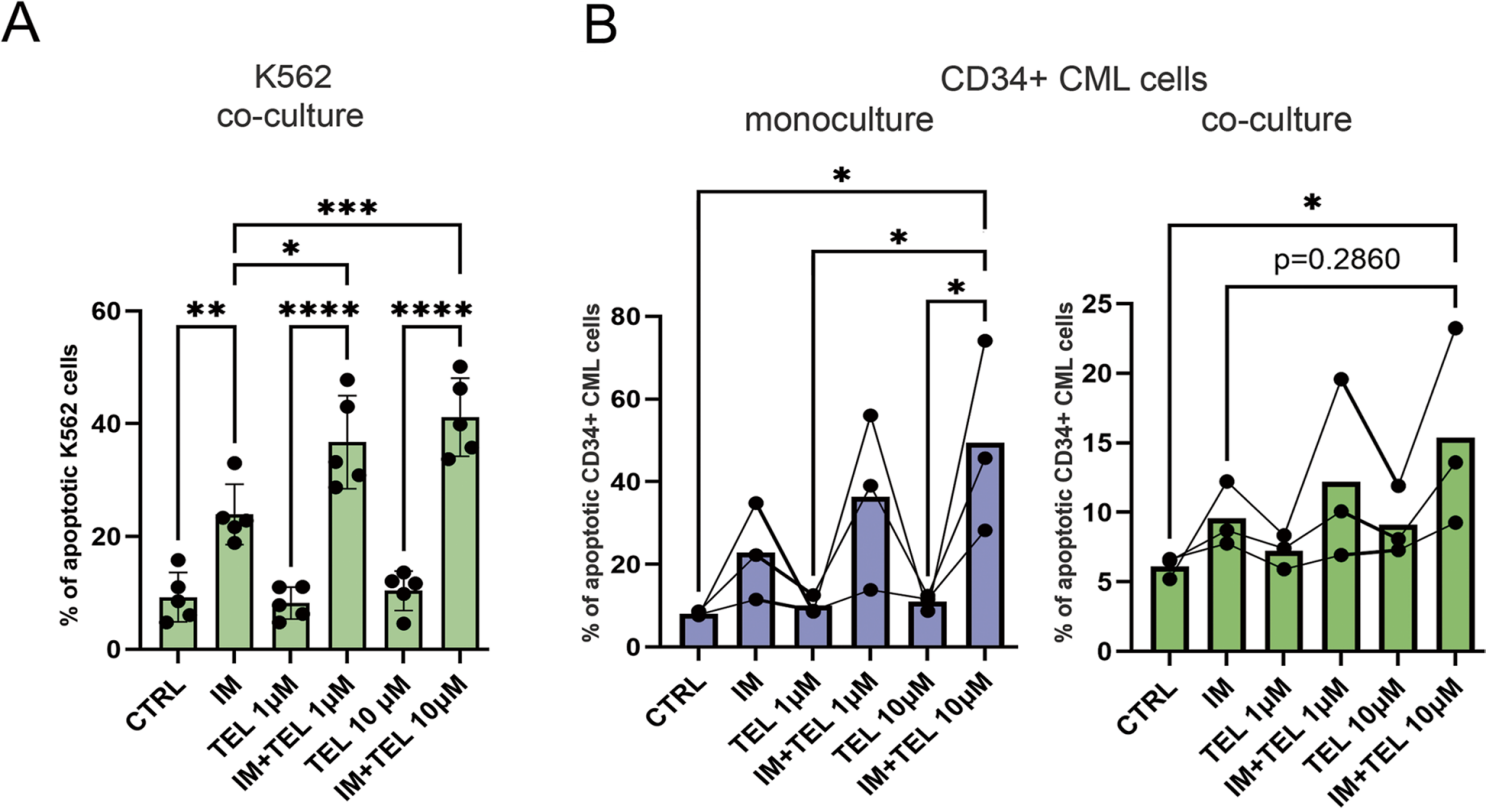
Targeting the TCA-related aerobic metabolism overcomes the stroma-mediated protection form imatinib.**A** Apoptosis detected in eFluor450-labeled K562 cells grown in co-culture with HS-5 cells. Cell were not treated (CTRL) or treated with 1 µM imatinib (IM), 1 µM or 10 µM telaglenastat (TEL) or combination of these two drugs (IM + TEL) for 48 h. Percentage of apoptotic leukemic cells was assessed by the Annexin V-7AAD cytometric assay, and eFluor450 + Annexin V-positive cells were counted as apoptotic. The data from *n* = 5 are shown as mean ± SEM. Values of *p* < 0.05 were considered to indicate statistical significance. *p < 0.05, **p < 0.01, ***p < 0.001. **B **Apoptosis detected in primary CD34 + CML cells grown in monoculture (left) or co-culture with HS-5 cells (right). Cells were not treated (CTRL) or treated with 1 µM imatinib (IM), 1 µM or 10 µM telaglenastat (TEL) or combination of these two drugs (IM + TEL) for 72 h. Percentage of apoptotic leukemic cells was assessed by the Annexin V-7AAD cytometric assay, and CD34 + Annexin V-positive cells were counted as apoptotic. The data from *n* = 3 are shown as mean ± SD. Values of *p* < 0.05 were considered to indicate statistical significance. *p < 0.05, **p < 0.01, ***p < 0.001

We found that telaglenastat used in combination with imatinib in leukemic cell line as well as in primary CD34 + cells from CML patients attenuated the stroma-mediated protection from imatinib (Fig. 7). This was visible as dose-dependent increased apoptosis in leukemic cells grown in co-culture treated with both drugs (IM + TEL) compared to the co-culture treated with imatinib alone or telaglenastat alone. This data showed that the combined treatment is efficient, however single treatments with either TKI or telaglenastat are not able to break the stroma-dependent resistance to apoptosis. The same trend was observed in primary CML cells, however more patients should be analyzed due to samples heterogeneity. Altogether this confirmed that the stroma-mediated increased Gln-TCA cycle activity is driver of the stroma-mediated protection from imatinib and potential therapeutic target, as well as indicated the potential of telaglenastat in combination with TKIs as pharmacological treatment to overcome the stroma-mediated resistance in leukemia.

Overall, we demonstrate here a novel, stroma-driven protective mechanism in leukemia, which is associated with the direct transfer of TCA-related proteins from stromal to leukemic cells, associated with shift towards non-glucose energy sources to feed TCA cycle. This direct transfer together with increased fuel of TCA with non-glucose substrates, enhances TCA cycle activity, leading to protection against metabolic drops after imatinib treatment. These changes consequently lead to increased metabolic plasticity and adaptation driven by the BMM, particularly active when leukemic cells are treated with imatinib. Our data also pinpoint these mechanisms as important factors in development of BMM-dependent resistance in chronic myeloid leukemia and potential targets for therapeutic intervention to eliminate leukemic cells from the BM niche.

## Discussion

The resistance of leukemia within the BMM has emerged as a significant clinical problem. Therefore, understanding the mechanisms underlying the stroma-driven protection is essential for developing effective therapies. This study reveals a novel role for the stroma-leukemia interactions, related to direct intercellular transfer of membrane vesicles together with active proteins to remodel metabolic fitness and plasticity in leukemia.

We found that direct horizontal transfer of stromal proteins that fuel the TCA cycle in leukemic cells promotes metabolic plasticity and survival in leukemia. The involvement of direct cell-cell connections via TNTs, due to the lack of a specific inhibitor, is typically verified using indirect methods including physical cell separation in line with imaging [5, 34–36]. Previously inhibitors of actin polymerization such as cytochalasin have been used to inhibit TNTs. However, due to actin cytoskeleton disruption, they have shown broad side effects and cytotoxicity, including disturbances of mitochondria dynamics and damage of mitochondrial network, disruption of cell division, motility and morphology [37], thereby impacting data interpretation. Therefore, we have not used those agents in our study. Instead, by using a trans-well system which physically prevented direct contact, as well as by incubating the leukemic cells with stromal conditioned medium, excluding the role of secreted factors, we confirmed the crucial role of direct stroma-leukemia connections in protein transfer, metabolic remodeling and stroma-driven protection from imatinib-induced apoptosis.

Mechanistically, the BM stroma transfers membrane vesicles with TCA cycle-related proteins and activates the TCA cycle in leukemia cells, together with increased TCA fuel by non-glucose Gln input, protection from oxidative stress, and the metabolic remodeling. This mediates the stroma-dependent metabolic plasticity and protection from imatinib effects such as metabolic decline and apoptosis. Even if stroma-mediated resistance and metabolic remodeling has been reported in other studies [2, 3, 25], such a direct stroma-driven mechanism fueling the TCA cycle has not been previously reported.

Recently, also direct horizontal transfer of mitochondria was proposed to be a potential protective mechanism related to immune evasion in cancers [38], tissue revitalization [39] and tumorigenic potential in mitochondrial DNA-deficient cancer cells [40]. Our data ruled out the oxidative phosphorylation of stromal mitochondria and the direct mitochondria transfer as the major mechanisms responsible for metabolic adaptation and plasticity caused by the stroma as well as resistance to imatinib. However, we cannot exclude that some of the stromal vesicles directly transferred together with proteins to leukemia cells have a mitochondrial origin and because of this contain mitochondrial proteins. This would be consistent with recent discoveries which showed that also mitochondria can generate specific extracellular vesicles (mitoEVs), which can transfer mitochondrial proteins to distant cells via secretion [41–43]. To explore this phenomenon, additional in-depth ultrastructure studies need to be performed. Nevertheless, independent of their origin, mitochondrial proteins need to be incorporated into the mitochondrial matrix. Our observation that the transferred proteins may contain a specific cleavable peptide localization sequence together with biological confirmation that the identified proteins can be transferred from stroma to leukemia strongly support the proposed mechanism.

The TCA cycle is a series of oxidative metabolic reactions that takes place in the mitochondrial matrix and generate energy via oxidative phosphorylation [44, 45]. This process utilizes either glucose, or non-glucose sources such as some amino acids or fatty acids, which increase the non-glucose respiratory capacity. Among non-glucose amino acids, glutamine emerges as an essential and major amino acid to fuel TCA, participating in TCA supplementation by intermediates, especially in cancer cells [28]. The metabolic analyses indicates that stroma promotes the shift towards the non-glucose respiratory capacity and the TCA oxidative metabolism with Gln input, without increased Warburg effect confirmed by low glucose uptake and low lactate secretion. This was particularly evident after imatinib treatment. Notably, such metabolic remodeling was reported in cancer cells and was associated with the survival and clinical resistance [46]. Consistent with increased TCA activity the generation of ROS occurs, which can cause cellular damage [47]. Therefore, to prevent oxidative stress and cytotoxic effects as a result of increased TCA and OXPHOS in leukemic cells, the presence of stroma led also to increased SOD2 expression, decreased ROS production and oxidation in leukemia cells.

In addition to its role in catabolism, the TCA cycle is also critical for the biosynthesis of various molecules, including amino acids, lipids, and nucleotides (anaplerotic function) [48]. Therefore, the TCA cycle also functions as a “key anabolic hub” supporting tumor growth. Because of all these functions, the ability of cancer cells to increase TCA activity strongly associates with cancer cells metabolic fitness, survival, disease progression and resistance to therapy [14, 15]. Here we present that the bone marrow stroma activates TCA cycle activity and function in leukemia, leading to protection from imatinib-induced apoptosis. However, the increased cell fitness and metabolic plasticity are strongly related and play together as a stroma-driven protective and prosurvival effect and it is not possible to separate them biologically.

The potential of our findings is strongly supported by clinical sc-RNA Seq data presented here, which showed strong correlation between the expression levels of genes encoding TCA-related proteins and resistance to imatinib and new generation TKIs in CML patients. Importantly, all TCA-related proteins and metabolites transferred from the stroma to leukemic cells were among the identified differential resistance-associated genes. This suggests that the contact with bone marrow stroma shifts cells towards the resistance phenotype and further supports our statement that these elements might serve as a novel potential therapeutic targets. Our data are also supported by other study in which the procancerogenic role of the TCA cycle has been reported [49–55], however without clear explanation of the mechanisms. Increased TCA activity and OXPHOS were indicated to promote the survival of CML cells [56], and interfering with the TCA cycle overcame LSC resistance [57]. Our findings indicate that these protective mechanisms may occur in leukemic cells when they are associated with the stroma and may protect against TKIs. Data from primary CD45+ CD34+ CML blasts support this conclusion as they show that stroma increases mitochondrial dependence and non-glucose capacity in leukemia, while simultaneously decreasing glucose dependence and glycolytic capacity. Also the cell-based phenotyping assays used to interrogate and characterize mitochondria show increased utilization of substrates related to TCA cycle. This is also present under imatinib treatment in BMM conditions, and is associated with increased viability and protection from apoptosis. This finding is consistent with the observation that AML leukemia cells depend on amino acid metabolism to fuel oxidative phosphorylation, which results in resistance [58, 59]. The pharmacological inhibition of amino acid metabolism reduced OXPHOS and induced apoptosis in these cells.

Our data indicate that the presence of stroma induces metabolic plasticity and fitness, associated with resistance, which are based on both, increased TCA activity and increased Gln input. Consistent with this, to verify the clinical potential of our findings we targeted the mechanism activated by the BMM with telaglenastat, to break the stroma-driven metabolic remodeling. Combination of telaglenastat with imatinib used in co-culture conditions attenuated the stroma-mediated protection and increased apoptosis of leukemic cells compared to treatment with imatinib or telaglenastat alone. The use of telaglenastat in combination with the first-line drug or radiosensitizer in anticancer therapy has already been reported [60, 61], and the phase I/II trials indicate clinical potential of telaglenastat [31–33]. Our data confirmed the potential of such therapeutic strategy to break the BM-mediated resistance to TKIs and to eliminate leukemic cells residing in the BMM.

## Conclusions

Altogether, we pinpointed direct cell-to-cell transfer of membrane vesicles together with proteins as a driver of metabolic plasticity in leukemia. To our knowledge, this was not reported before. Stroma-dependent remodeling of metabolism has already been noted; however, other mechanisms have been proposed [62–64]. One of the study correlated expression of nestin in stroma as important for AML resistance associated with increased OXPHOS and TCA [64]. These data, even if not discussed that way by the authors, may indirectly support our observations as nestin has been proposed as a TNT marker [65, 66].

Finally we conclude that direct transfer of TCA cycle-related proteins from stromal to leukemic cells is a novel protective mechanism driven by the bone marrow stromal microenvironment. This mechanism fuels the activity of the TCA cycle, contributing to metabolic plasticity, increased metabolic fitness and protection from imatinib effects in leukemia. This observation is fully supported by the single-cell RNA Seq data indicating that expression of TCA genes strongly correlates with resistance to imatinib and new generation tyrosine kinase inhibitors. Targeting the TCA metabolism [67], as a precision medicine in leukemia, will be of considerable interest. Ongoing research indicates potent therapeutic strategies and several small molecule inhibitors are being tested, partly in trials [67–70]. Our findings presented here imply that targeting the TCA cycle-related mechanism, including the Gln input to fuel TCA in leukemia, by telaglenastat, prevents building the stroma-mediated metabolic plasticity and may be a prospective direction for therapeutic development to overcome the BMM-mediated resistance to TKIs in leukemia.

## Supplementary Information

Supplementary Material 1.

Supplementary Material 2.

## Data Availability

The data that contribute to the findings of this study are available within the article and included in Supplementary files. The MS proteomics data have been deposited to the ProteomeXchange Consortium via the PRIDE [https://www.ebi.ac.uk/pride] partner repository with the dataset identifier PXD013504. Further data supporting the findings of this study are available from the corresponding author upon reasonable request.

## Abbreviations

7-AAD: 7-aminoactinomycin D
AAO: Amino acid oxidation
AML: Acute myeloid leukemia
ATP: Adenosine triphosphate
BC: Blast crisis
BM: Bone marrow
BMM: Bone marrow microenvironment
BSA: Bovine serum albumin
CCCP: Carbonyl cyanide m-chlorophenylhydrazone
CD: Cluster of differentiation
CENCAT: Cellular energetics through noncanonical amino acid tagging
CM: Conditioned medium
CML: Chronic myeloid leukemia
CoA: Coenzyme A
CP: Chronic phase
CuAAC: Copper(I)-catalyzed azide-alkyne cycloaddition
ddC: 2'-3'-dideoxycytidine
DLST: Dihydrolipoamide s-succinyltransferase
DMEM: Dulbecco's Modified Eagle Medium
DMR: Deep molecular response
ECAR: Extracellular acidification rate
EDTA: Ethylenediaminetetraacetic acid
EV: Extracellular vesicle
FAD/FADH2: Flavin adenine dinucleotide
FAO: Fatty acid oxidation
FBS: Fetal bovine serum
FCCP: Carbonyl cyanide-p-trifluoromethoxyphenylhydrazone
FLT3: Fms like tyrosine kinase 3
GFP: Green fluorescent protein
Gln: Glutamine
GM-CSF: Granulocyte-macrophage colony-stimulating factor
HPG: Homopropargylglycine
IL: Interleukin
IMDM: Iscove's modified dulbecco's medium
LC-MS: Liquid chromatography-mass spectrometry
LSC: Leukemic stem cell
MFI: Mean fluorescence intensity
mitoEVs: Mitochondrial extracellular vesicles
MMR: Major molecular response
MS: Mass spectrometry
mtDNA: Mitochondrial DNA
NAD/NADH: Nicotinamide adenine dinucleotide
NADP/NADPH: Nicotinamide Adenine Dinucleotide Phosphate Hydrogen
OCR: Oxygen consumption rate
OXPHOS: Oxidative phosphorylation
PLL: Poly-L-lysine
PSM: Peptide spectrum macthes
SCENITH: Single-cell energetic metabolism by profiling translation inhibition
SCF: Stem cell factor
scRNA-seq: Single-cell RNA sequencing
SILAC: Stable-isotope labeling of amino acids in cell culture
SRC: Spare respiratory capacity
TCA cycle: Tricarboxylic acid cycle
THPTA: Tris-hydroxypropyltriazolylmethylamine
TKI: Tyrosine kinase inhinitor
TNT: Tunneling nanotube
TPO: Thrombopoietin
