# Correction: Stroma-driven horizontal transfer of TCA-related proteins mediates metabolic plasticity and Imatinib resistance in chronic myeloid leukemia

**DOI:** 10.1186/s12964-026-02677-7

**Published:** 2026-01-20

**Authors:** Piotr Chroscicki, Nikodem Kasak, Dorota Dymkowska, Laura Turos-Korgul, Dominik Cysewski, Vira Chumak, Dawid Stepnik, Monika Kusio-Kobialka, Agata Kominek, Magdalena Lebiedzinska-Arciszewska, Alicja Krop, Joanna Szczepanowska, Mariusz Wieckowski, Tomasz Stoklosa, Krzysztof Zablocki, Katarzyna Piwocka

**Affiliations:** 1 Laboratory of Cytometry, Nencki Institute of Experimental Biology, Polish Academy of Sciences, Warsaw, Poland; 2 Laboratory of Cellular Metabolism, Nencki Institute of Experimental Biology, Polish Academy of Sciences, Warsaw, Poland; 3 Clinical Research Centre, Medical University of Bialystok, Bialystok, Poland; 4 Laboratory of Mitochondrial Biology and Metabolism, Nencki Institute of Experimental Biology, Polish Academy of Sciences, Warsaw, Poland; 5 Laboratory of Genetics, University Clinical Center, Medical University of Warsaw, Warsaw, Poland; 6 Department of Tumor Biology and Genetics, Medical University of Warsaw, Warsaw, Poland

**Correction: Cell Communication and Signaling 24, 7 (2026)**

**https://doi.org/10.1186/s12964-025-02564-7**

Following publication of the original article [1], The author has requested to replace Fig. 7 as Fig. 5 is duplicated and shown also as Fig. 7 which was mistakenly processed by the typesetting team.

The original article [1] has been corrected.
