# Supplementary material for: Stroma-driven horizontal transfer of TCA-related proteins mediates metabolic plasticity and imatinib resistance in chronic myeloid leukemia
Source: Cell Commun Signal. 2025 Dec 2;24:7. doi: 10.1186/s12964-025-02564-7 (PMC12777312; doi:10.1186/s12964-025-02564-7)
Supplement: Supplementary file 1 — Supplementary Material 1. [file 12964_2025_2564_MOESM1_ESM.pdf]

Supplementary Material

Stroma-driven horizontal transfer of proteins related to the Glutamine-TCA cycle mediates metabolic plasticity and imatinib resistance in chronic myeloid leukemia

Piotr Chroscicki[1],*, Nikodem Kasak[1],*, Dorota Dymkowska[2], Laura Turos-Korgul[1], Dominik Cysewski[3], Vira Chumak[1], Dawid Stepnik[1], Marta Kolba[1], Monika Kusio-Kobialka[1], Agata Kominek[1], Magdalena Lebiedzinska-Arciszewska[4], Alicja Krop[5], Joanna Szczepanowska[2], Mariusz Wieckowski[4], Tomasz Stoklosa[5,6], Krzysztof Zablocki[2], Katarzyna Piwocka[1,#]

1. Laboratory of Cytometry, Nencki Institute of Experimental Biology, Polish Academy of Sciences, Warsaw, Poland
2. Laboratory of Cellular Metabolism, Nencki Institute of Experimental Biology, Polish Academy of Sciences, Warsaw, Poland
3. Clinical Research Centre, Medical University of Bialystok, Bialystok, Poland
4. Laboratory of Mitochondrial Biology and Metabolism, Nencki Institute of Experimental Biology, Polish Academy of Sciences, Warsaw, Poland
5. Laboratory of Genetics, University Clinical Center, Medical University of Warsaw, Poland
6. Department of Tumor Biology and Genetics, Medical University of Warsaw, Poland

- These authors contributed equally to this work

# Correspondence: k.piwocka@nencki.edu.pl

Table 1
Patients characteristics

	Age	Sex	CML stage at diagnosis	Resistance / identified mutations (NGS)	% blasts in blood
**P181**	55	M	CML-CP	Primarily resistance ABL1 E255K mutation	1% blasts
**P371**	30	M	CML-CP	No NGS diagnostics	80% IG (immature cells)
**P372**	51	K	CML-CP	No NGS diagnostics	81G/L leukocytosis
**P356**	52	K	CML-BC	MSH2 and WT1 mutations	27% blasts

For the experiments, CD34+ cells were isolated from PBMC using an EasySep human CD34+ selection cocktail. The CD34+ population has been then verified by flow cytometry and showed 98-99% purity. All data presented in the manuscript are from the experiments performed on the pure fraction of CD34+ CML cells.

Supplementary Figures

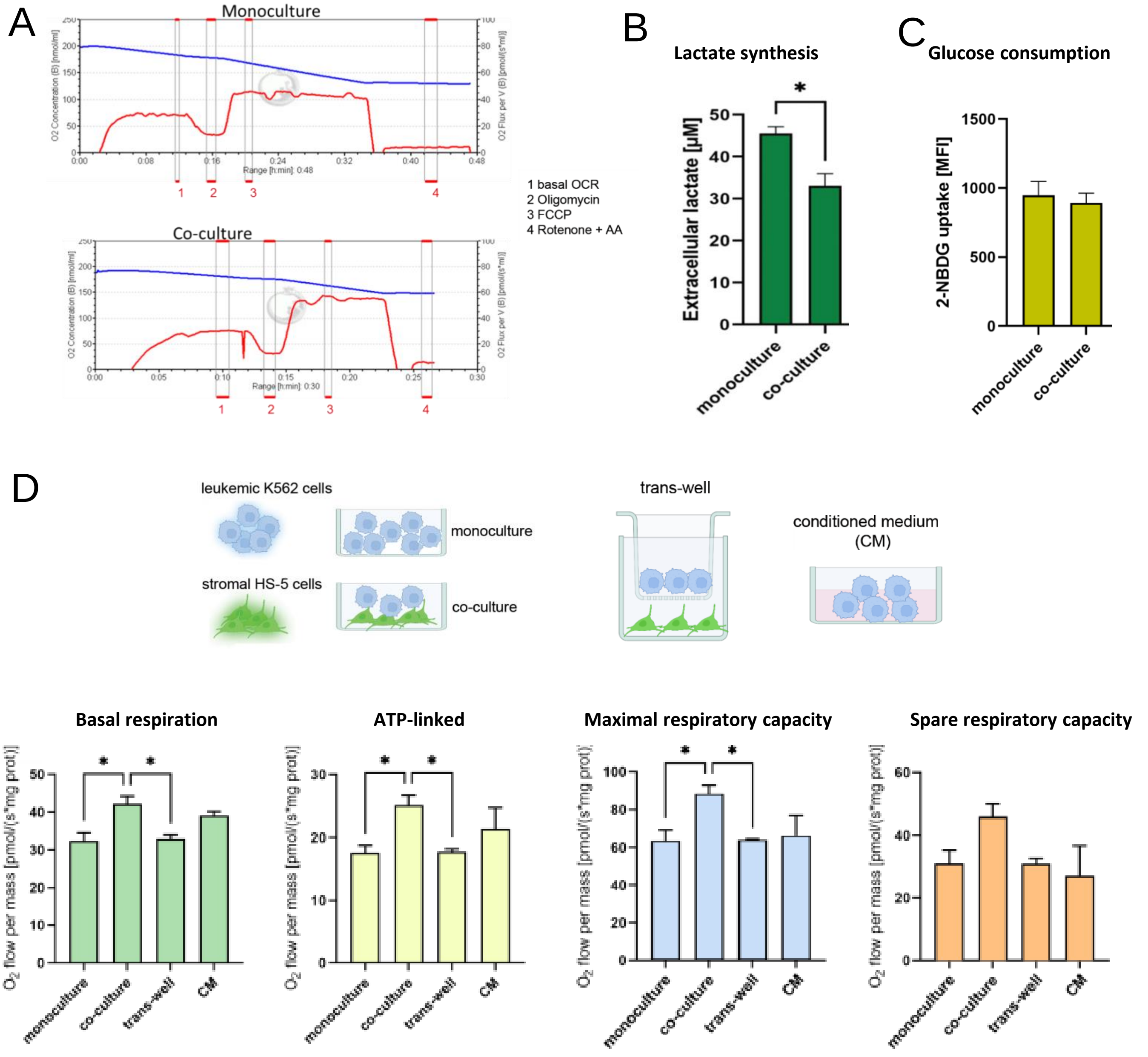

Supplementary Figure 1

A. The oxygen consumption rates measured by respirometry method (Oroboros) in leukemia cells grown in monoculture or co-culture for 24 hours, treated with Oligomycin, CCCP, rotenone and antimycin A. The red line shows oxygen consumption. 1 - basal respiration, 2 - ATP-synthase independent respiration, 3 - maximal respiration, 4 - non-mitochondrial oxygen consumption. Representative oxygraphs are shown. The red curve represents respiration rate, the blue curve depicts oxygen flux per volume. Horizontal red lines in the upper part of each diagram represent timeframes of data collection. **B**. Lactate production in leukemic cells cultured for 24 hours in monoculture or co-culture. Mean values (n=4 ± SD) are presented. **C**. Glucose consumption measured in leukemic cells cultured for 24 hours in monoculture or co-culture with glucose analoque 2-NBDG for 20 minutes. Mean values (n=4 ± SD) are presented. **D.** Metabolic profiles assessed by the respirometry analysis in monoculture, co-culture, as well as co-culture in the trans-well system or leukemic cells incubated with conditioned medium (CM) obtained from the stromal cells. The graphs show O2 flow Mean values (n=4 ± SD) are presented. Unpaired parametric t-test was used, p-values <0.05 were considered as statistically significant; *p < 0.05, **p < 0.01, ***p < 0.001, ****p<0.0001.

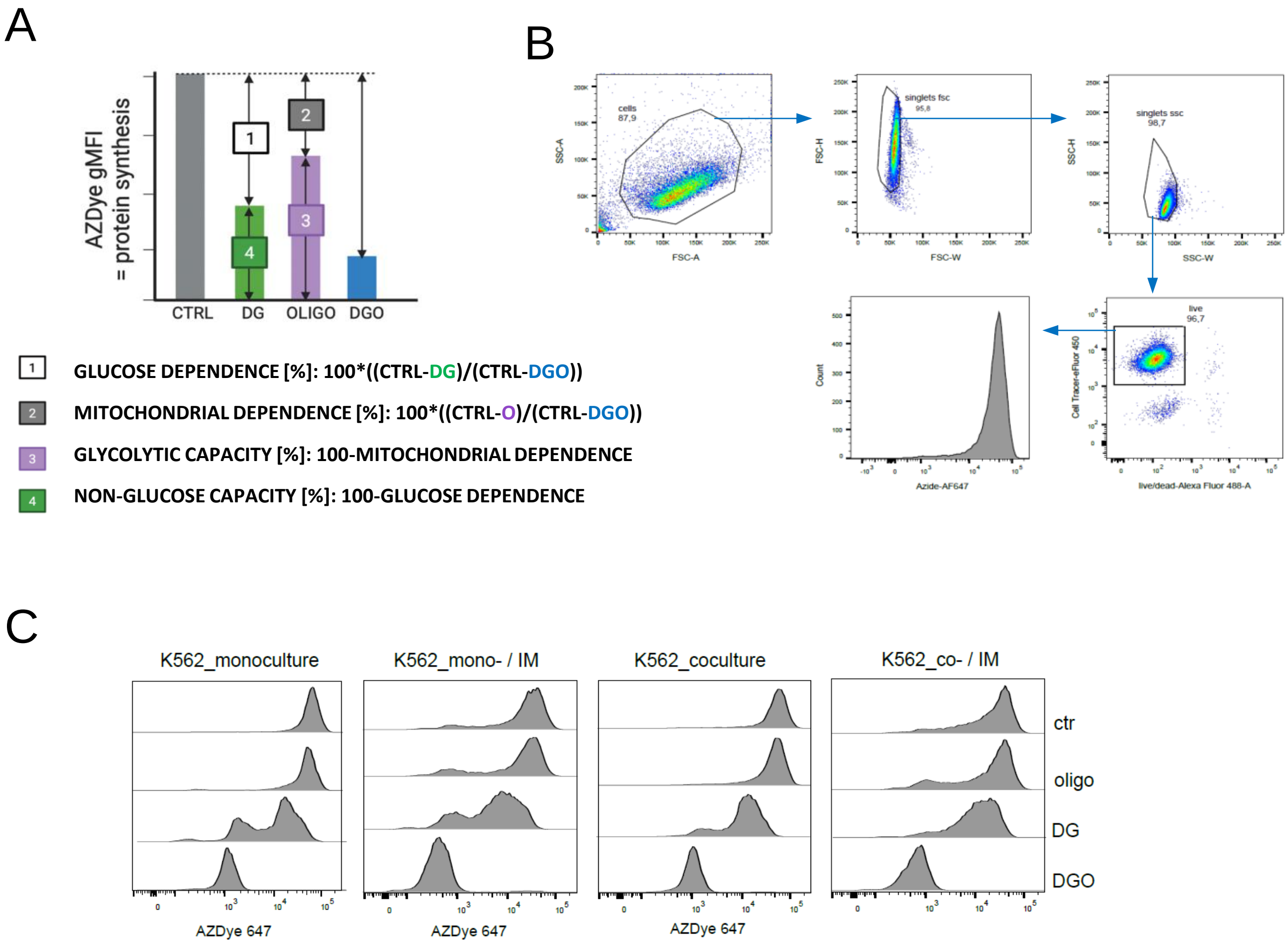

Supplementary Figure 2

A. Calculations used in the CENCAT method, using gMFI values of Azide-647 fluorescence**. B.** The gating strategy for the flow cytometry analysis using CENCAT. The K562 cells were sequentially gated on cells (FSC-A/SSC-A) and single events to exclude doublets (FSC-A/FSC-H and SSC-A/SSC-H), viable cells (live/dead Alexa Fluor 488) and cell tracer eFluor450, followed by detection of the fluorescence of azide-Az647. Typical dot plots and histograms are presented. CML K562 cells grown for 24 hours in monoculture or co-culture with stromal HS-5 cells, without treatment or after treatment with imatinib for 24 hours. K562 cells were labeled with a fluorescent dye (eFluor450) before the co-culture for better separation. **C**. Representative histograms of fluorescent Azide-647 measured by BD Fortessa flow cytometer in control (Ctr) sample, or samples treated with 2-Deoxy-Glucose (DG), Oligomycin (O) or combination (DGO) in leukemic cells grown in monoculture (M) or co-culture (CO), untreated or treated with imatinib (IM) are shown.

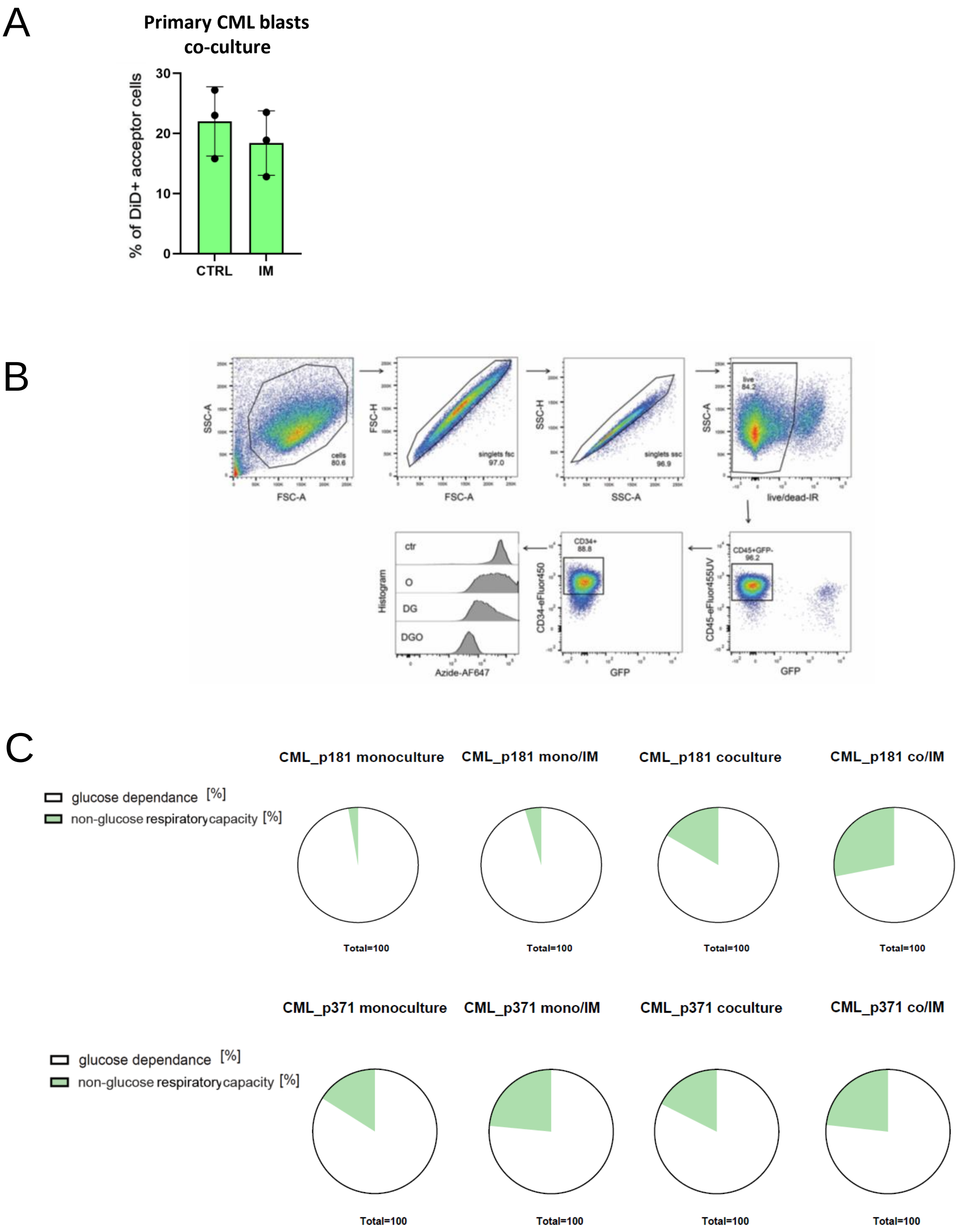

Supplementary Figure 3

A. Transfer of vesicles from stromal to primary CML blast cells detected by flow cytometry based on hCD45+ expresion. Vesicles were stained by DiD in stromal donors and detected after 24 hours of co-culture in leukemic recipients. Percentage of DiD+ leukemic cells is shown. **B**. The gating strategy for flow cytometry analysis using CENCAT method. The primary CD34+ cells isolated from two CML patients in chronic phase (p181 ans p371) were sequentially gated on cells (FSC-A/SSC-A) and single events to exclude doublets (FSC-A/FSC-H and SSC-A/SSC-H), viable cells (live/dead Alexa Fluor 488), CD45 (eFluor455UV) and CD34 (eFluor450), followed by detection of the fluorescence of azide-Az647. Typical dot plots are presented. CML cells were grown for 24 hours in monoculture or co-culture with stromal HS-5 cells, without treatment or after treatment with imatinib for 24 hours. **C**. Pie charts representing the metabolic profile of glucose dependence versus non-glucose respiratory capacity measured by the CENCAT in leukemic cells from CML patients (p181 – CML-ChP; p471 – CML_ChP) grown in monoculture (M) or co-culture (CO), untreated or treated with imatinib (IM).

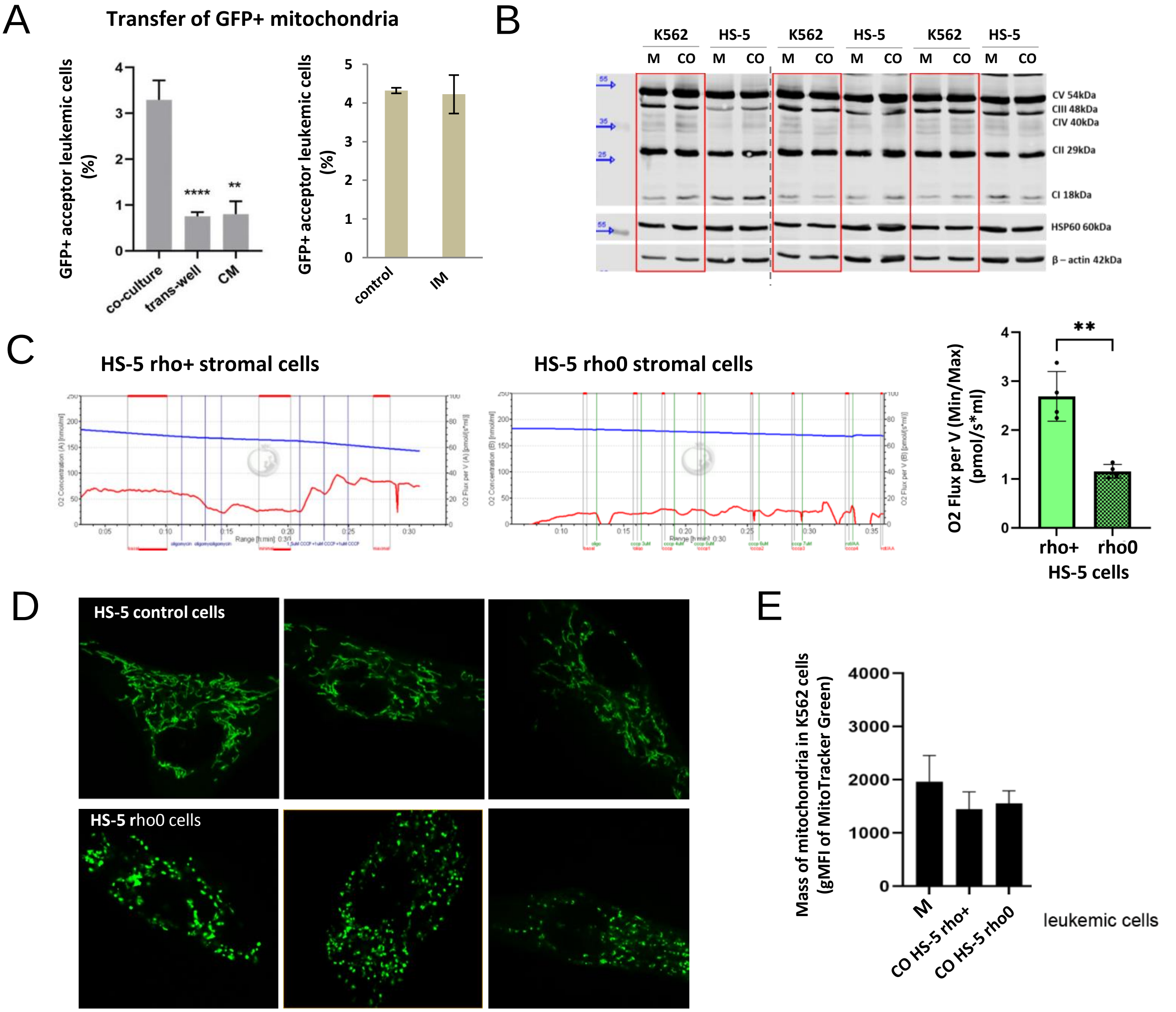

Supplementary Figure 4

A. Transfer of GFP+mitochondria from stromal to leukemic cells assessed by flow cytometry in leukemia cells (fluorescently labeled with proliferation dye eFluor647), grown in co-culture with HS-5 stromal cells expressing GFP-tagged mitochondria, in co-culture in the trans-well system or in monoculture supplemented with conditioned medium (CM) from stromal cells (left graph) or untreated or treated with 2 μM imatinib for 24 hours (right graph). The percentage of GFP+acceptor cells shows leukemic cells which received stromal mitochondria. Mean values (n=3 ± SEM) are presented. Mean values (n=3 ± SEM) are presented. Statistical analysis was performed in trans-well and CM conditions, in comparison with co-culture conditions. Unpaired parametric t-test was used, p-values <0.05 were considered as statistically significant; *p < 0.05, **p < 0.01, ***p < 0.001, ****p<0.0001. **B.** The levels of respiratory subunit proteins estimated by Western Blotting in either HS-5 or K562 cells growing in mono- (M) or co-culture (CO). Data from three sets of experiments are presented. Dotted line indicates membranes cutting sites. Uncropped membranes are presented in the separate file „Raw data" as part of the Supplemental materials. **C**. Oxygraphs showing oxygen consumption in control HS-5 cells and rho0 HS-5 cells. Cells were treated with Oligomycin, CCCP, and rotenone. Red line shows oxygen consumption. Representative experiment. Right graph - the statistical analysis of oxygen consumption. Mean values (n=4 ± SEM) are presented. **D**. Visualization of organization of mitochondrial network of control and Rho0 cells expressing mitochondrial GFP protein. The representative confocal images are presented. **E.** Mitochondrial mass estimated by flow cytometry in K562 cells stained with MitoTracker Green. Leukemic control cells were grown in monoculture (K562 M) or co-culture (CO) with either HS-5 with metabolically active mitochondria (CO HS-5 rho+) or dysfunctional rho0 stromal cells (CO HS-5 rho0). Mean geomean values (gMFI) (n=3 ± SEM) are presented. Unpaired parametric t-test was used, p-values <0.05 were considered as statistically significant; *p < 0.05, **p < 0.01, ***p < 0.001, ****p<0.0001.

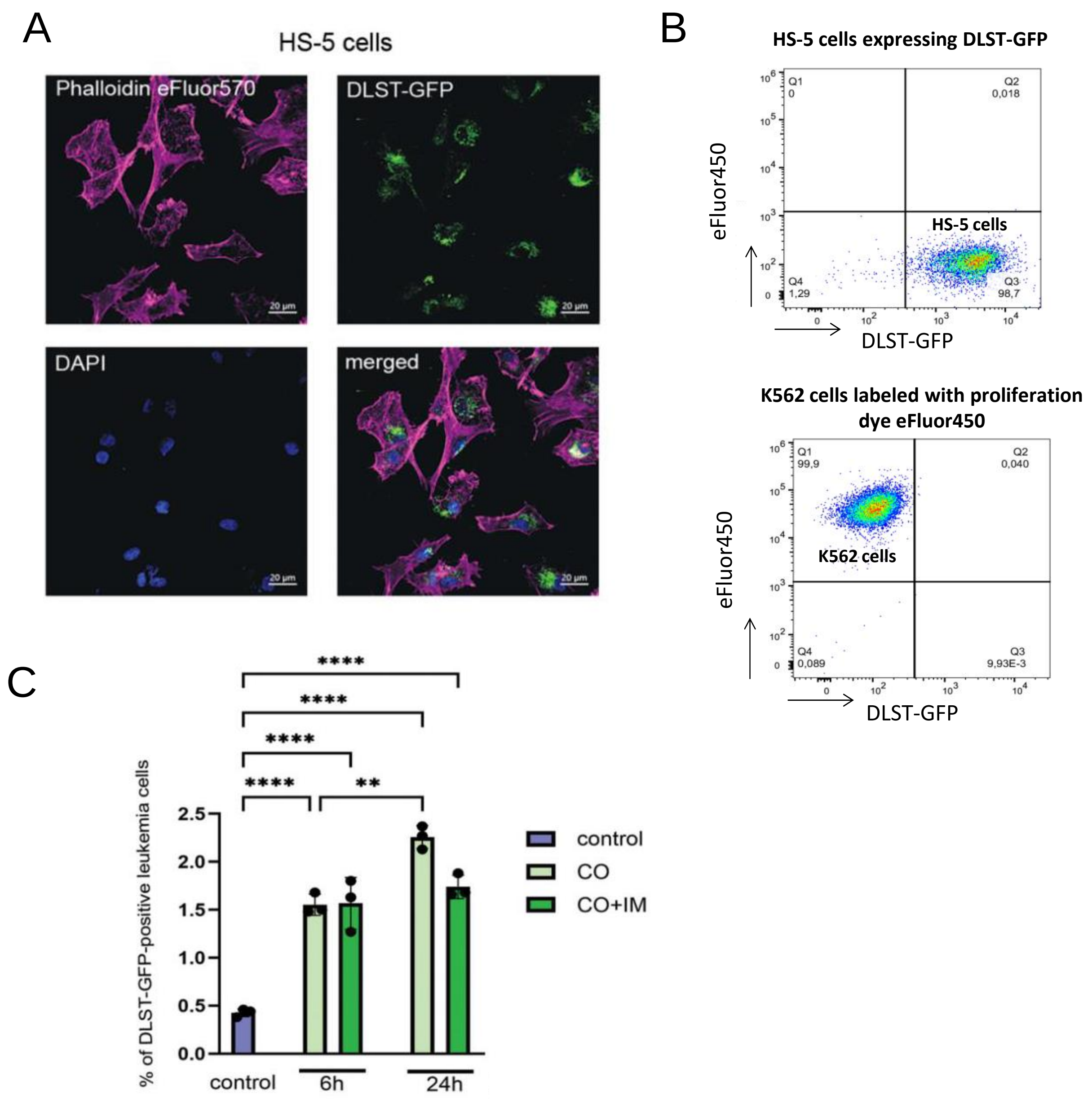

Supplementary Figure 5

A. Representative immunofluorescence of HS-5 cells expressing DLST-GFP protein (green), additionally stained with Phalloidin eFluor670 (purple) and DAPI to visualize nuclei (blue). Single fluorescence images and merge signals are presented. Scale bar 20 µM. **B**. Flow cytometry analysis of HS-5 cells expressing the DLST-GFP protein and K562 cells labeled with a fluorescent dye (eFluor450) before the co-culture. Upper panel - HS-5 cells (DLST donors) express DLST-GFP protein (96.7%) and are negative to eFluor450. Lower panel - K562 cells are GFP-negative and eFluor450-positive (99.9%). Representative dot plots are shown. Analysis was performed using BD LSR Fortessa flow cytometer. **C**. The percentage of DLST-GFP -positive leukemic recipients indicating transfer from stromal donors to leukemic recipient cells estimated by flow cytometry in control cells or co-culture (CO) after 6 and 24 hours, without or with imatinib (IM) treatment. Mean values (n=3 ± SD) are presented. For statistical analysis, all co-culture conditions were compared to monoculture, 6 hour treatment to 12 hours as well as untreated to imatinib-treated. Unpaired parametric t-test was used, p-values <0.05 were considered as statistically significant; *p < 0.05, **p < 0.01, ***p < 0.001, ****p<0.0001.

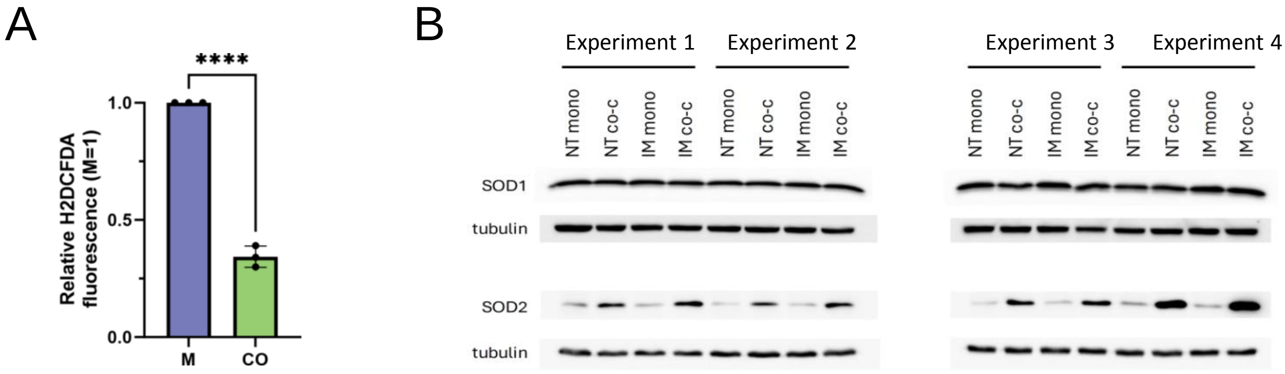

Supplementary Figure 6

A. ROS levels measured by the H2DCFDA assay in leukemic cells growing for 24 hours in monoculture (M) or co-culture (CO). Cells were incubated with cell-pearmeable ROS probe and fluorescence was detected by flow cytometry. The values in monoculture are normalized to 1. Mean values (n=3 ± SD) are presented. Unpaired parametric t-test was used, p-values <0.05 were considered as statistically significant; *p < 0.05, **p < 0.01, ***p < 0.001, ****p<0.0001. **B**. Western blot analysis of the levels of SOD1 and SOD2 proteins in K562 cells in monoculture (mono) or co-culture (co-c), untreated (NT) or treated 24 hours with imatinib (IM). Tubulin was used as a leading control. Data from four independent experiments are shown.
