# Supplementary figures and images for: Stroma-driven horizontal transfer of TCA-related proteins mediates metabolic plasticity and imatinib resistance in chronic myeloid leukemia
Source: Cell Commun Signal. 2025 Dec 2;24:7. doi: 10.1186/s12964-025-02564-7 (PMC12777312; doi:10.1186/s12964-025-02564-7)

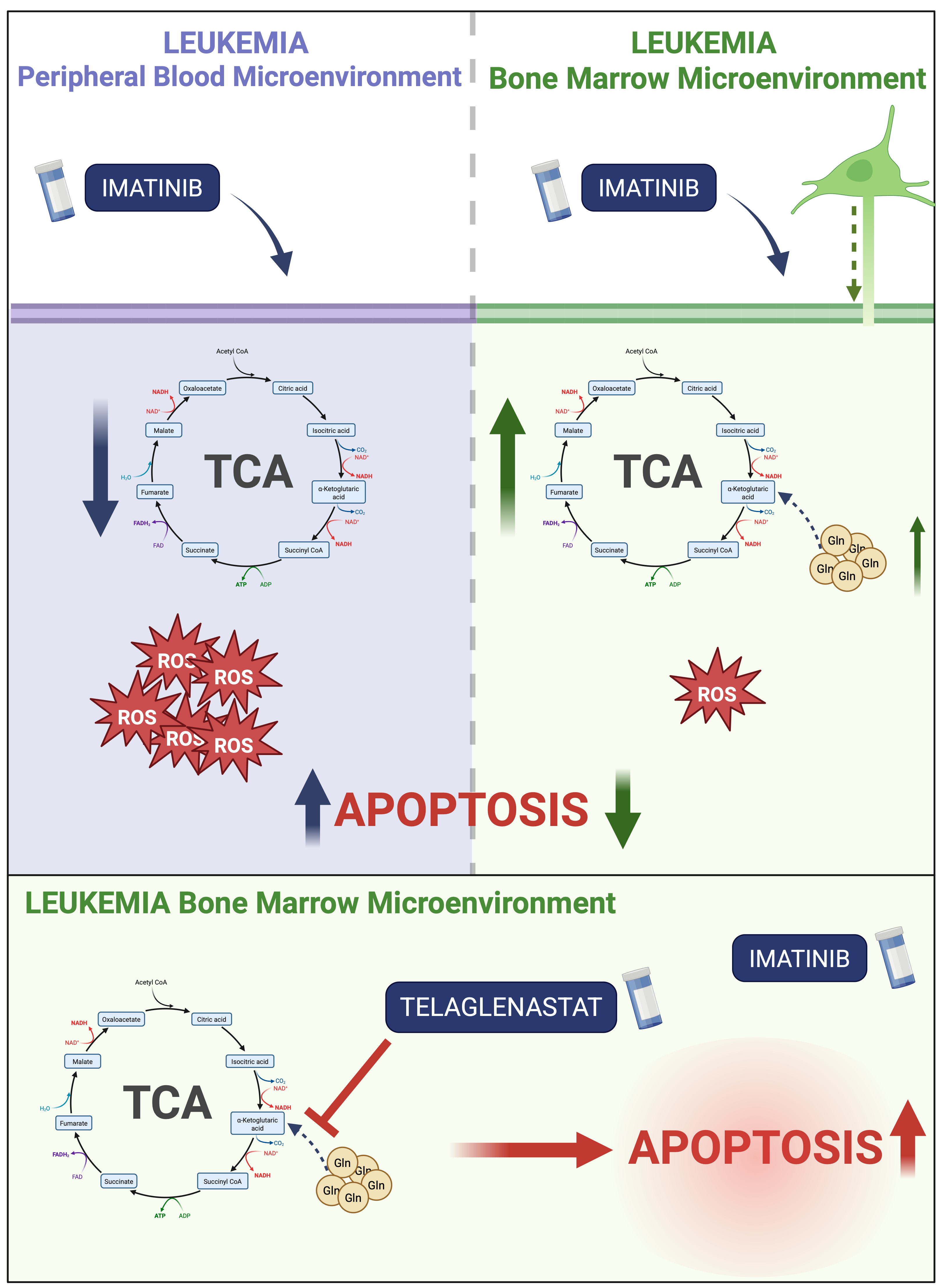

Supplement: Supplementary file 2 — Supplementary Material 2. [file 12964_2025_2564_MOESM2_ESM.png]
